# Wearable Motion Sensor Device to Facilitate Rehabilitation in Patients With Shoulder Adhesive Capsulitis: Pilot Study to Assess Feasibility

**DOI:** 10.2196/17032

**Published:** 2020-07-23

**Authors:** Yu-Pin Chen, Chung-Ying Lin, Ming-Jr Tsai, Tai-Yuan Chuang, Oscar Kuang-Sheng Lee

**Affiliations:** 1 Institute of Clinical Medicine National Yang Ming University Taipei Taiwan; 2 Department of Orthopedic Surgery Wan Fang Hospital Taipei Medical University Taipei Taiwan; 3 Department of Orthopedic Surgery School of Medicine, College of Medicine Taipei Medical University Taipei Taiwan; 4 Department of Rehabilitation Sciences Faculty of Health and Social Sciences The Hong Kong Polytechnic University Hung Hom China (Hong Kong); 5 Department of Orthopedic Surgery Puli Christian Hospital Nantou Taiwan; 6 Department of Medical Research Taipei Veterans General Hospital Taipei Taiwan; 7 Department of Orthopedics China Medical University Hospital Taichung Taiwan

**Keywords:** motion sensor, adhesive capsulitis, rehabilitation, home-based exercise, telerehabilitation, telehealth, telemonitoring

## Abstract

**Background:**

Adhesive capsulitis (AC) of the shoulder is a common disorder that painfully reduces the shoulder range of motion (ROM) among middle-aged individuals. Although physical therapy with home-based exercises is widely advised to restore ROM in the treatment of AC, clinical results vary owing to inconsistent patient compliance.

**Objective:**

In this study, we aimed to verify the feasibility of a treatment model that involves applying a wearable motion sensor device to assist patients conduct home-based exercises to improve training compliance and the accuracy of exercises, with the ultimate goal of improving the functional recovery of patients with AC.

**Methods:**

The motion sensor device was comprised of inertial measurement unit–based sensors and mobile apps for patients and physicians, offering shoulder mobility tracing, home-based exercise support, and progress monitoring. The interrater reliability of shoulder mobility measurement using the motion sensor device on 10 healthy participants and 15 patients with AC was obtained using an intraclass correlation coefficient analysis and compared with the assessments performed by two highly experienced physicians. A pilot prospective control trial was then carried out to allocate the 15 patients with AC to two groups: home-based exercise group and motion sensor–assisted rehabilitation group. Changes in active and passive shoulder ROM, pain and functional scores, and exercise completion rates were compared between the groups during a treatment period of 3 months.

**Results:**

Shoulder ROM, as measured using the motion sensor device, exhibited good to excellent reliability based on the comparison with the measurements of two physicians (intraclass correlation coefficient range, 0.771 to 0.979). Compared with patients with AC in the home-based exercise group, those in the motion sensor–assisted rehabilitation group exhibited better shoulder mobility and functional recovery and a higher exercise completion rate during and after 3 months of rehabilitation.

**Conclusions:**

Motion sensor device–assisted home-based rehabilitation for the treatment of AC is a useful treatment model for telerehabilitation that enhances the compliance of patients through training, thus improving functional recovery. This helps overcome important obstacles in physiotherapy at home by providing comprehensible and easily accessible exercise instructions, enhancing compliance, ensuring the correctness of exercise, and monitoring the progress of patients.

## Introduction

Adhesive capsulitis (AC) of the shoulder, which occurs in approximately 2% to 5% of the general population, is an idiopathic, progressive, and painful restriction of the active range of motion (aROM) and passive range of motion (pROM) [1,2]. AC typically affects patients older than 50 years, and involvement of both shoulders is noted in 20% to 30% of cases [1,2]. Although symptomatic improvement tends to occur naturally within years even with minimal treatment [3], approximately 50% of patients experience pain or some mild restriction of movement, and 11% experience some residual disability even several years after the resolution of their other symptoms [4,5]. Although AC is a common condition, high-quality evidence of successful treatment methods for AC has not yet been obtained [6]. The well-accepted standard treatment for AC mostly involves physical therapy and home exercises to restore ROM [7,8]. Evidence suggests that, compared with less frequent self-exercise in a home setting or joint mobilization sessions in a hospital setting, regular and daily self-exercise in a home setting could contribute to greater improvement in shoulder ROM and a shorter duration of symptoms [9].

Despite realizing the importance of daily physiotherapy, including mobilization and strength exercises, clinicians have achieved varied outcomes in patients who are trained in a home-based exercise program [10]. Challenges that affect the effectiveness of home-based exercises for AC may include training compliance and exercise correctness. Patients with AC do not maintain their training frequency and duration at home because the prescribed exercise programs are typically not followed without the constant supervision of a physiotherapist. Failure to incorporate exercises into daily life is the main form of noncompliance, as reported in up to 60% of patients whose treatment plan included home-based exercise [11]. Whether patients can correctly perform exercises at home after initial instruction by physiotherapists is also a concern [12]. Therefore, the development of methods to improve compliance and exercise correctness for patients with AC is worthwhile to maximize the effectiveness of home-based physiotherapy.

Techniques for detecting bodily motions are extensively used in health care to monitor and rehabilitate disabled patients. With the evolution of sensing and body area network technologies, wearable rehabilitation technology has opened up the possibility of independent training, which has advantages over traditional rehabilitation services [13]. Inertial measurement units (IMUs), including accelerometers and gyroscopes, have been extensively used in technology-assisted rehabilitation, with sufficient efficacy [14-16]. However, despite the potential of using IMU-based sensors in neurorehabilitation and for treating musculoskeletal impairments [13,17,18], few such sensors have been used in clinical studies, especially those involving physiotherapy for AC.

Through this study, we tested wearable IMU-based sensors that integrated with interactive mobile apps using wireless telecommunication technology to enable physiotherapists to monitor the progress of patients with AC who were conducting home-based self-exercise and improve their training compliance. We hypothesized that the use of a wearable IMU-based motion sensor device could help patients perform home-based exercises correctly and increase their motivation and compliance regarding training, thereby improving functional outcomes.

## Methods

### Motion Sensor Device

A wearable motion sensor device (BoostFix wearable self-training kit, COMPAL Electronics Inc, Taipei, Taiwan) was used (see Multimedia Appendix 1). The motion sensor device was comprised of wearable IMU-based sensors that record the angular shoulder motion of a patient, a mobile app termed Patient App for use by the patient, and a mobile app termed Doctor App for use by a qualified health care professional.

#### Wearable IMU–Based Sensors

**Figure 1:**
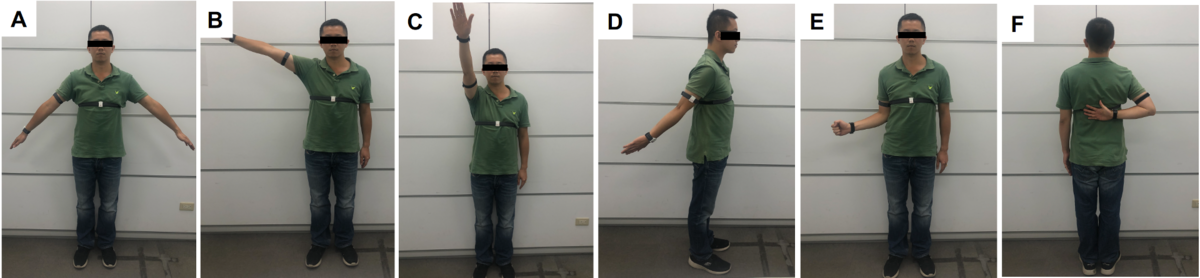
Angular measurement of the shoulder with motion sensors worn on the body for (A) calibration, (B) shoulder abduction, (C) shoulder flexion, (D) shoulder extension, (E) shoulder external rotation, (F) shoulder internal rotation.

The IMU-based sensors were comprised of 6-axis microelectromechanical systems that included accelerometers and gyroscopes. These devices collect information about the angular motion of the shoulder of interest. Three sensors are required for angular measurements of the shoulder. In this study, they were strapped to the sternum, distal third of the lateral upper arm approximately 5 cm above the lateral epicondylar of the humerus bone, and dorsal wrist (Figure 1). Three sensors were calibrated in position A once the measurement began (Figure 1A). The initial calibration process involved placing the sensor on a horizontal fixture to measure the offset for each axis, to align the initial difference for each sensor. The calibration process involved obtaining the initial value of the sensor for relative offset correction instead of correcting the deviation caused by incorrect sensor placement. In addition, the process entailed identifying the original offset of the sensors to each other and applying the zero setting using an algorithm.

**Figure 2:**
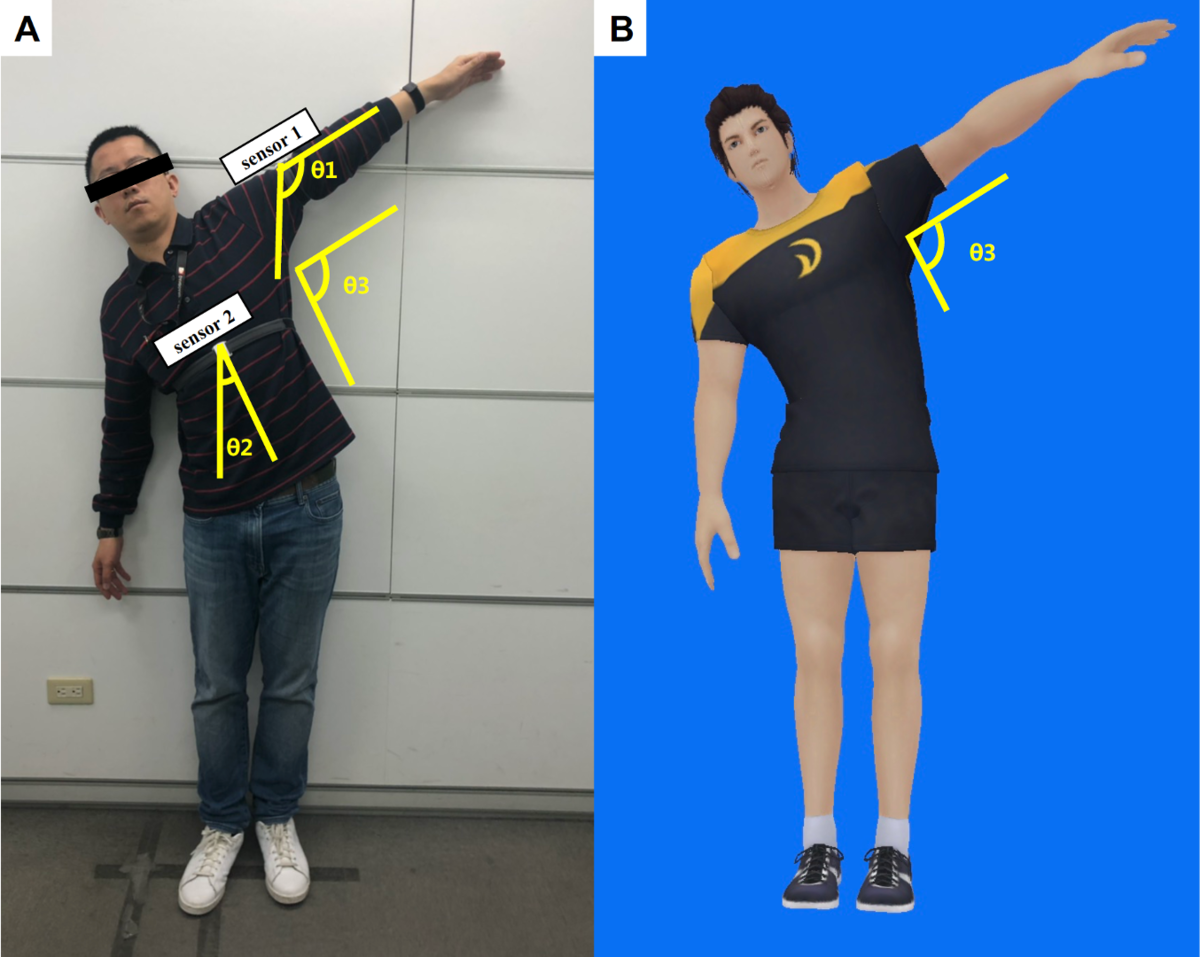
Dual-sensor system simulates the motion of (A) patients with adhesive capsulitis performing shoulder abduction using a (B) 3-dimensional avatar. θ1: angular motion reported by the sensor on the upper arm; θ2: axial body tilting angle reported by the sensor on the sternum; θ3: shoulder abduction angle (ie, θ1 - θ2).

Dual-sensor systems were used in the motion sensor device to accurately record the shoulder ROM angle. Using the sensors on the sternum as a reference datum point, the raw data from each sensor can be converted into a quaternion algorithm to convert the relative angle changes of the sensors on the upper arm and wrist into a 3-dimensional (3D) motion of the shoulder structure. Although one sensor can provide the angular change with the gyroscopes on one plane of motion, dual-sensor systems can provide relative angular changes in shoulder movements between the arm and axial body (by compensating for the angle of the body tilting relative to the ground), which was superior for eliminating the error in the shoulder ROM measurement attributed to body tilting from a one-sensor system, especially for patients with AC and painful shoulder movement (Figure 2). Additionally, repeated angular measurements with the dual-sensor system on simulated shoulder motion was validated as highly accurate. This is referred to in Multimedia Appendix 2.

#### Mobile Phone App for Patients

**Figure 3:**
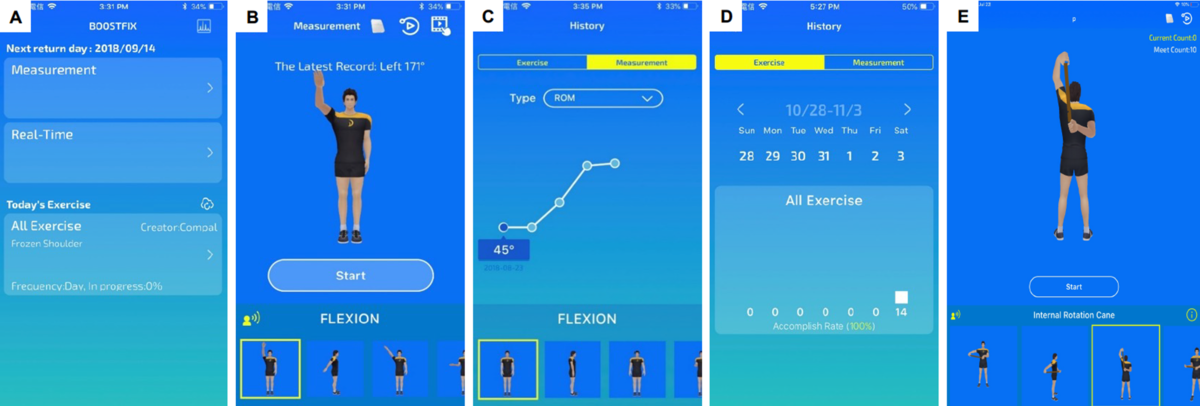
Examples of screens on the mobile app for patients, including the (A) functional introduction, (B) shoulder mobility measurement, (C) historical records of shoulder range of motion, (D) historical records of daily exercise completion rate, (E) daily home-based exercise tasks.

**Figure 4:**
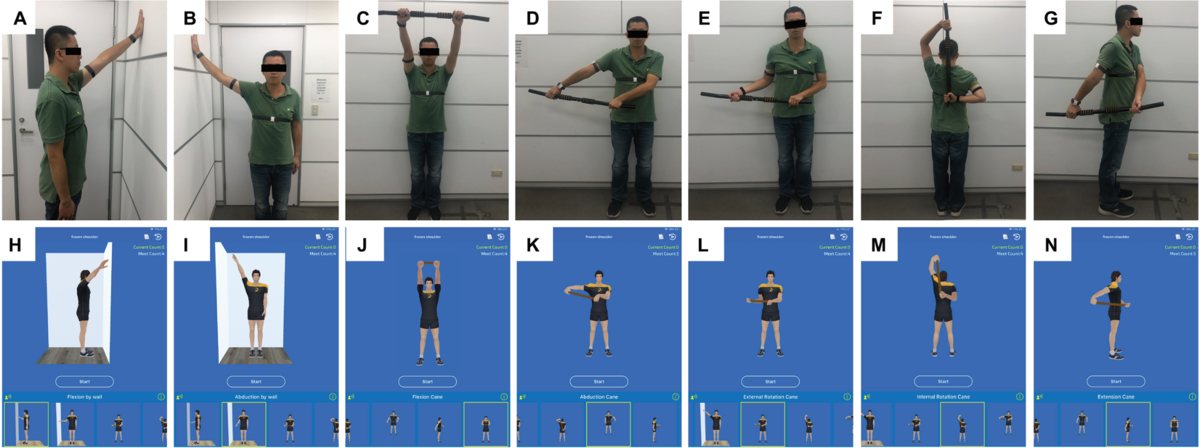
Sets of home-based shoulder exercises displayed on the mobile app and demonstrated by participants: (A, H) forward wall walking stretch; (B, I) lateral wall walking stretch; (C, J) cane stretch for shoulder flexion; (D, K) cane stretch for shoulder abduction; (E, L) cane stretch for shoulder external rotation; (F, M) cane stretch for shoulder internal rotation; (G, N) cane stretch for shoulder extension.

To design the mobile app for patients with AC for use on mobile phones, a cocreation process was used. The app has modes for measuring shoulder mobility, generating historical records of angular measurements and exercise completion rates, and providing daily shoulder exercises with detailed instructions (Figure 3). The mobile app determines shoulder ROM in all directions by calculating the relevant information regarding the angle measurement reported by the 3 wearable sensors (Figures 2B and 3B). The Patient App provides 7 sets of home-based shoulder exercises for training, including a forward wall walking stretch, lateral wall walking stretch, and cane stretch for shoulder flexion, extension, abduction, internal rotation, and external rotation (Figure 4). The mobile app assigns each set of exercises as a “daily task” assigned by the supervising physiotherapist or physicians and provides a demonstration using a 3D avatar (Figure 3E). The app mirrors the user’s shoulder movement during the exercise using angular information collected from the worn motion sensors. Each user has an account in the Patient App to access his or her records, which includes daily progress and the completion rate of the daily exercises (Figures 3C and 3D).

#### Mobile App for Physiotherapists and Physicians

**Figure 5:**
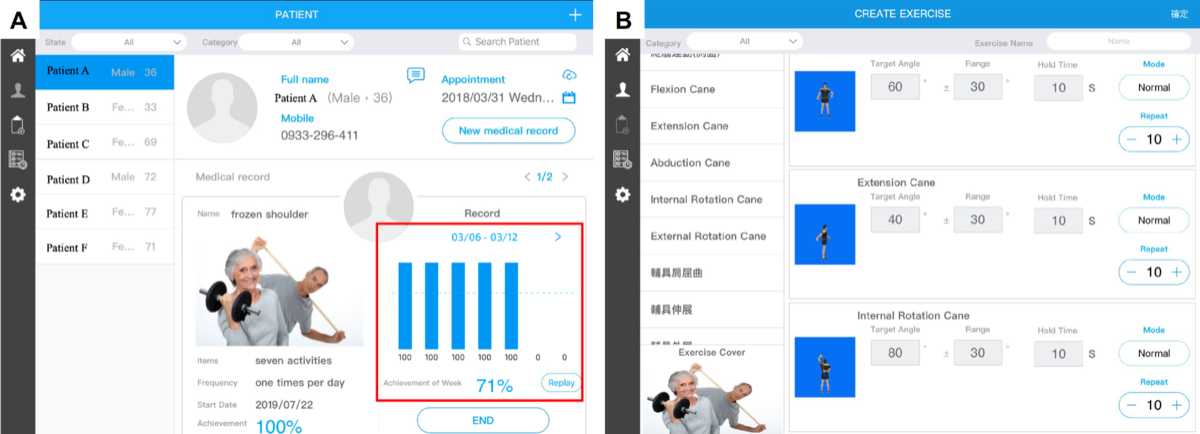
Examples of screens on the mobile app for physiotherapists and physicians, including the (A) daily and weekly exercise completion rate of each patient displayed with patient information and (B) doctor-assigned daily exercises with specified targeted angles, holding times, and numbers of repetitions.

The Doctor App was designed for physiotherapists and physicians for use on a mobile tablet. This app provides each patient’s information, including the latest shoulder ROM measurements and exercise completion rates for the previous week (Figure 5A). Physiotherapists and physicians can assign personalized daily home-based exercises with adjustable targeted angles, numbers of repetitions, and holding times for each patient based on the angular status of the affected shoulder (Figure 5B). Physiotherapists/physicians can directly communicate with the patients by sending text messages through the Doctor App.

### Study Protocol

This study included two investigations. The first evaluated the reliability of measurements of shoulder ROM using the motion sensor device, and the second confirmed the effectiveness of using the motion sensor device for the rehabilitation training of patients with AC. The Ethical Committee of Taipei Medical University approved the entire protocol and instrumentation (TMU-JIRB N201708048). All participants consented to participation in the study and the publication of data.

#### Reliability of Shoulder ROM Measurements Using a Motion Sensor Device

From January 2017 to August 2017, two groups of participants — 10 healthy participants and 15 patients with a confirmed diagnosis of AC — were prospectively enrolled for measurement of shoulder angular motion.

Volunteers aged 20 to 70 years were included in the group of healthy participants. Participants who reported discomfort or limited ROM of the shoulder within the preceding year were excluded. Patients were included in the AC group who (1) were aged between 40 and 70 years, (2) had spontaneous onset of a painful stiff shoulder and marked loss of active and passive global shoulder motion, (3) had reported local shoulder pain that was frequently present either over the anteromedial aspect of the shoulder extending distally into the biceps region or over the lateral aspect of the shoulder extending into the lateral deltoid region, and (4) had symptoms for at least 3 months, with normal findings on anteroposterior and axillary radiographs of the glenohumeral joint. Patients were excluded if they (1) had pathological findings of rotator cuff tear on physical examination (eg, abduction weakness and positive dropped arm test); (2) had glenohumeral osteoarthritis upon radiographic evaluation; (3) had clinical evidence of severe cervical spine disease; (4) had a history of severe trauma to the shoulder; (5) received a local corticosteroid injection or any physiotherapeutic intervention to the affected shoulder in the 3 months preceding the study start date; (6) had a history of inflammatory joint disease, infection, stroke, or thyroid diseases that affected the shoulder; or (7) were unwilling to undergo an intervention or participate in the trial.

For angle measurements, aROM and pROM for flexion, extension, abduction, and internal and external rotation at a neutral position for both shoulders of healthy participants and for the affected shoulder of the patients with AC were assessed. The shoulder ROM in all directions for each participant was first assessed using the motion sensor device. Two examiners (one well-trained physiotherapist and one highly experienced orthopedic surgeon) who were blinded to the angle measurements by the motion sensor device measured shoulder ROM in all directions using a universal goniometer.

Each participant stood straight, and scapular rotation was permitted to measure abduction, flexion, and extension (Figures 1B, 1C, 1D). External rotation was measured in a neutral position with the shoulder abducted, the elbow flexed at 90°, and the forearm in a neutral position; the angle between the long axis of the forearm and the sagittal plane of the trunk was measured as external rotation (Figure 1E). Internal rotation was determined by having the participants reach up the back to the highest point along the midline (Figure 1F). Six levels of internal rotation were defined according to the areas that the participant’s hand reached, as follows: interscapular region (T7), T12 vertebra, waist (L3), lumbosacral junction, buttock, and lateral thigh. A high reach corresponded to better internal rotation. The aROM was measured by instructing participants to move their arm as far as they could, and the pROM was measured as the examiner moved the subject’s arm to its mechanical limit or a limit imposed by pain.

#### Effectiveness of Motion Sensor Device for Rehabilitation Training of Patients with AC

The 15 patients with confirmed diagnoses of AC were allocated to 2 intervention groups: 7 patients to the home-based exercise group and 8 patients to the motion sensor–assisted rehabilitation group. The patients were allocated according to their willingness to use, and familiarity with, mobile apps. The patients in both groups received 3 months of rehabilitation.

Patients with AC in the home-based exercise group received comprehensive instructions on the daily shoulder exercises from an experienced orthopedic surgeon before beginning the exercise program. The information included instructions for home-based exercises; a description of frozen shoulder; and advice on sleep, posture, and pain relief. Home-based exercises included Codman’s or pendulum exercises (circumduction) and passive stretching exercises, including “wall walking stretch exercises” and “cane stretch exercises” (Figure 4). Further information regarding the standard protocol of the home-based exercises is available in Multimedia Appendix 3. The patients were instructed to perform these exercises within the painless range, maintain the maximal achievable angle for at least 10 seconds in each exercise, and repeat each exercise at least 10 times daily. All patients were instructed to follow the standard home-based exercise protocol, but individualized modification was permitted based on health professionals’ judgment. In practice, an orthopedic surgeon reviewed the progress of each patient every month. They gave advice and modified the exercise program based on the patient’s compliance and AC status. Therefore, if a patient had poor performance on a specific direction of shoulder ROM after the exercise program of the previous month (eg, on shoulder abduction), the orthopedic surgeon could make adjustments to the next month’s exercise protocol by increasing the daily exercise frequency to 15-20 times and the holding time to 15-20 seconds per session for the “lateral wall walking stretch” and “cane stretch for shoulder abduction” exercises.

All patients in the motion sensor–assisted rehabilitation group received detailed instructions on operating the motion sensor device and Patient App on their own mobile phones before they began the rehabilitation program. The aforementioned orthopedic surgeon assigned the standard protocol of daily home-based shoulder exercises to each patient via the Patient App with individualized modifications according to the patient’s performance. The completion rate of these daily tasks was calculated using the app for each patient and sent to the Doctor App over the internet, enabling the supervisor to immediately discuss the progress of each patient with them. In addition, patients in the motion sensor–assisted rehabilitation group were individually assigned each set of stretching exercises with the target angle on the mobile app by the orthopedic surgeon based on the patients’ previous shoulder angular performance (Figure 5B). For example, if the patient had 100° shoulder abduction before starting the shoulder exercise program, the target angle for the “lateral wall walking stretch” and “cane stretch for shoulder abduction” would be assigned as 120° with an acceptable range of 30°. Achievement of the target angle of the assigned exercise could be recorded as a successful count for calculating the daily exercise completion rate on the mobile app (Figure 3D). In this manner, the patient could therefore stretch the involved shoulder to the maximal limit of the angle on each attempt, thereby effectively maximizing the training effect of stretching exercises.

### Outcome Measurement

Basic demographic data, including age, gender, and educational status, were collected for each patient with AC through face-to-face interviews. For all patients, shoulder aROM and pROM in all directions of the affected shoulder were assessed and recorded by the motion sensor device at baseline and each month for 3 months following the initiation of the rehabilitation program. All patients received and completed questionnaires that allowed relevant metrics to be evaluated at baseline and at the 1-month, 2-month, and 3-month follow-ups by an independent physiotherapist who was not involved in treating any patients and was blinded to the treatment allocation. This questionnaire was based on a pain severity visual analog scale (VAS) and the Quick Disabilities of the Arm, Shoulder, and Hand (qDASH) questionnaire. The monthly exercise completion rate, as reported by all participants and by the motion sensor devices in the motion sensor–assisted rehabilitation group, was recorded every month.

### Instruments

#### Measurement of Shoulder Function

The self-reported qDASH, consisting of 11 items, was developed to measure the relevant physical function and symptoms of patients with upper limb disorders [19]. The Chinese version of qDASH has excellent reliability (Cronbach α=.818, intraclass correlation coefficient [ICC]=0.907) [20]. A higher qDASH score indicates poorer shoulder function.

#### Measurement of Pain

A VAS is an instrument that is regularly used to measure the intensity of pain [21]. A researcher asked the patients, “On a scale of 0 to 10, how severe has been the worst pain that you have experienced within the last week?” This question was repeated in the follow-up sessions.

#### Measurement of Exercise Completion Rate

The exercise completion rate reported by the motion sensor device was calculated by dividing the number of exercises completed daily by the number of assigned exercise tasks daily. The monthly exercise completion rate was the average of the daily exercise completion rates over an entire month. The patient-reported exercise completion rate in each month was obtained by asking each patient, “On a scale of 0%-100%, what was your average exercise completion rate in the previous month?”

### Statistical Analysis

The ICC was used to calculate the interrater reliability between the shoulder aROM and pROM outcomes measured by the motion sensor device and the two experienced examiners. The ICC was obtained by conducting a 2-way analysis of variance in a random effects model. ICCs >0.8 and >0.9 were considered to indicate good and excellent reliability, respectively [22]. The ICC was further used to determine the minimal clinically important difference (MCID) in a distribution-based method. Specifically, the standard error of the measurement (SEM) was first computed using the following equation: SEM = SD of the baseline measures × 0√1 − ICC; then, the MCID was computed as follows: SEM × 1.96 × √2 [23,24].

In the second part of the study, in which the home-based exercise and motion sensor–assisted rehabilitation groups were compared, the Mann-Whitney *U* test was used to compare two independent, ordinal groups*,* regardless of the normal distribution of the metric of interest owing to small sample sizes. The Wilcoxon signed-rank test was used for within-group comparisons of the dependent variables at follow-up. The MCID computed from the first part of the study was used to illustrate whether the changes in aROM and pROM were meaningful. Along with descriptive statistics concerning participant characteristics, generalized estimating equations were used to compare the two groups with respect to improvements in several outcomes (including aROM, pROM, VAS, and qDASH). All generalized estimating equations were analyzed using the restricted maximum likelihood estimation. They all controlled for the effects of time during follow-up. SPSS 23.0 (IBM Corp, Armonk, NY) was used for all data analyses. *P*≤.05 was considered to indicate statistical significance.

## Results

Regarding the first part of the study on the reliability of measurement by the motion sensor device, Table 1 presents the results for shoulder aROM and pROM in all directions for healthy participants and for patients with AC, as obtained by the motion sensor device and the two examiners.

**Table 1:** Active and passive ranges of motion of the shoulder measured by different examiners.

Examiner		Abduction (°), mean (SD)		Flexion (°), mean (SD)					Extension (°), mean (SD)				External rotation (°), mean (SD)				Internal rotation (level)^a^		
		Active	Passive	Active			Passive		Active		Passive		Active		Passive		Active		Passive
**Healthy participants (10 patients, 20 shoulders)**																			
	Examiner 1	167.4 (9.1)	173.2 (5.2)	161 (8.4)			164.9 (5.6)		61.5 (7.9)		74.7 (8.7)		74.7 (6.6)		85.6 (4.6)		13^b^, 6^c^, 1^d^		18^b^, 2^c^
	Examiner 2	167.1 (8.5)	171.6 (6.7)	161.8 (9.9)			166 (7.1)		59.3 (5.4)		71.3 (8.0)		74.3 (8.7)		84.6 (4.8)		11^b^, 9^c^		17^b^, 3^c^
	Motion sensor device	162.4 (9.1)	171.7 (7.3)	156.6 (9.4)			162.9 (6.4)		56.2 (7.9)		69.1 (10.8)		77.6 (9.9)		86.2 (6.2)		15^b^, 5^c^		19^b^, 1^c^
**Patients with adhesive capsulitis (15 patients, 15 shoulders)**																			
	Examiner 1	117.6 (18.1)	128.9 (18.1)	130.7 (13.8)			141.8 (10.5)		56.7 (11.7)		65.7 (9.5)		49.3 (11.6)		61.3 (12.1)		1^b^, 2^c^, 12^d^		3^b^, 7^c^, 5^d^
	Examiner 2	118.8 (17.4)	129.5 (18.3)	132 (14.2)			144.9 (9.4)		56.3 (9.1)		64.7 (9.3)		51.4 (10.8)		60.1 (11.0)		1^b^, 3^c^, 11^d^		3^b^, 7^c^, 5^d^
	Motion sensor device	118.7 (17.8)	131.7 (18.7)	133.7 (13.2)			146.7 (11.4)		55.7 (9.8)		63.9 (8.5)		49.5 (11.1)		59.6 (12.2)		1^b^, 2^c^, 11^d^, 1^e^		3^b^, 7^c^, 5^d^

^a^Reported at the internal rotation level.

^b^Interscapular (T7).

^c^T12 vertebra.

^d^Waist (L3).

^e^Lumbosacral junction.

Table 2 shows whether the interrater reliability for the measurement of shoulder aROM and pROM in all directions, except for shoulder extension, as assessed by the motion sensor device and the two examiners was good or excellent (ICC range 0.899-0.979). The interrater reliability of the measurement of active and passive shoulder extension was fair to good (aROM: 0.771; pROM: 0.799).

**Table 2:** Interobserver reliability between angle measurements obtained by different examiners.

Values	Abduction		Flexion			Extension			External rotation			Internal rotation		
	Active	Passive		Active	Passive		Active	Passive		Active	Passive		Active	Passive
ICC^a^ among examiners	0.971	0.979		0.952	0.899		0.771	0.799		0.951	0.960		0.914	0.966
95% lower limit	0.950	0.964		0.918	0.832		0.640	0.680		0.916	0.931		0.855	0.941
95% upper limit	0.984	0.989		0.974	0.944		0.867	0.885		0.973	0.978		0.952	0.981

^a^ICC: intraclass correlation coefficient.

In the second part of the study on the effectiveness of the motion sensor device in rehabilitation training, one patient in the motion sensor–assisted rehabilitation group was excluded from the analysis owing to progressive shoulder pain and newly developed shoulder abduction weakness 1 month after rehabilitation. The patient received a subsequent diagnosis of a full-thickness rotator cuff tear based on shoulder magnetic resonance imaging. Therefore, the comparison was of 7 patients in the motion sensor–assisted rehabilitation group and 7 patients in the home-based exercise group. Table 3 presents the patient demographics, which did not differ significantly at baseline between the two groups.

**Table 3:** Comparison of patient demographics between groups.

Demographic characteristics		Motion sensor–assisted rehabilitation group (n=7)	Home-based exercise group (n=7)	*P* value
**Lesion side, n (%)**				
	Left	3 (42.9)	4 (57.1)	.59
	Right	4 (57.1)	3 (42.9)	
Age (years), mean (SD)		53.0 (6.2)	56.1 (13.3)	.35
**Gender, n (%)**				
	Male	4 (57.1)	5 (71.4)	.58
	Female	3 (42.9)	2 (28.6)	
**Education, n (%)**				
	Senior high	2 (28.6)	4 (57.1)	.28
	Bachelor’s degree and higher	5 (71.4)	3 (42.9)	
**Symptom duration (months), n (%)**				
	3-6	4 (57.1)	3 (42.9)	.59
	6-12	3 (42.9)	4 (57.1)	
**Range of motion, mean (SD)**				
	Active abduction (**°**)	122.4 (15.7)	113.1 (20.3)	.48
	Passive abduction (**°**)	126.9 (16.9)	131.6 (17.1)	.65
	Active flexion (**°**)	138.4 (14.4)	127.9 (11.0)	.28
	Passive flexion (**°**)	146.4 (15.7)	146.7 (7.3)	.75
	Active extension (**°**)	56.4 (12.6)	54.1 (5.0)	.95
	Passive extension (**°**)	63.9 (11.0)	63.1 (6.6)	.95
	Active external rotation (**°**)	52.4 (11.6)	49.0 (9.7)	.48
	Passive external rotation (**°**)	61.7 (14.0)	58.0 (11.9)	.52
	Active internal rotation (**°**)^a^	1^b^, 4^c^, 2^d^	1^b^, 2^c^, 4^d^	.16
	Passive internal rotation (**°**)^a^	2^b^, 4^c^, 1^d^	1^b^, 4^c^, 2^d^	.67
VAS^e^ score, mean (SD)		5.3 (1.3)	6.1 (1.8)	.37
qDASH^f^, mean (SD)		30.6 (18.1)	23.3 (7.2)	.90

^a^Reported at the internal rotation level.

^b^Interscapular (T7).

^c^T12 vertebra.

^d^Waist (L3).

^e^VAS: visual analogue scale.

^f^qDASH: Quick Disabilities of the Arm, Shoulder, and Hand questionnaire.

After 3 months of follow-up, patients in the motion sensor–assisted rehabilitation group exhibited a significant improvement from baseline in terms of shoulder aROM and pROM in all directions, pain score, and qDASH (Table 4). By contrast, patients in the home-based exercise group exhibited significant improvements only in aROM in shoulder abduction, aROM and pROM in shoulder extension, and VAS score (Table 5). Additionally, when the improvements in the motion sensor–assisted rehabilitation and home-based exercise groups were compared, the motion sensor–assisted rehabilitation group had achieved better and faster meaningful changes than the home-based exercise group.

**Table 4:** Improvement in parameters compared with baseline in the motion sensor–assisted rehabilitation group.

Parameters		Baseline	1 month	*P* value	2 months	*P* value	3 months	*P* value	MCID^a^
**Abduction (°), mean (SD)**									
	Active	122.4 (15.7)	148.1 (17.5)	.04	154.1 (11.9)	.02	156.7 (9.9)	.02	5.99
	Passive	126.9 (16.9)	152.9 (17.1)	.03	157.6 (10.7)	.02	161.6 (10.3)	.02	4.63
**Flexion (°), mean (SD)**									
	Active	138.4 (14.4)	149.1 (11.4)	.02	151.0 (5.4)	.04	159.6 (8.5)	.02	6.78
	Passive	146.4 (15.7)	162.0 (11.9)	.02	164.7 (5.6)	.03	170.1 (8.1)	.02	7.14
**Extension (°), mean (SD)**									
	Active	56.4 (12.6)	62.7 (12.6)	.06	65.9 (10.8)	.03	73.6 (11.9)	.03	11.15
	Passive	63.9 (11.0)	69.3 (12.2)	.11	78.1 (11.4)	.03	84.3 (6.4)	.02	11.36
**External rotation (°), mean (SD)**									
	Active	52.4 (11.6)	66.4 (15.4)	.02	64.7 (14.8)	.02	64.6 (18.8)	.04	5.88
	Passive	61.7 (14.0)	67.6 (18.6)	.20	71.9 (15.2)	.03	76.7 (15.1)	.02	4.44
**Internal rotation (level)^b^, mean (SD)**									
	Active	1^c^, 5^d^, 1^e^	1^f^, 4^c^, 2^d^	.01	3^f^, 2^c^, 2^d^	.04	3^f^, 2^c^, 2^d^	.04	—^g^
	Passive	1^f^, 3^c^, 3^d^	2^f^, 4^c^, 1^d^	.08	3^f^, 4^c^	.06	3^f^, 4^c^	.06	—^g^
VAS^h^ score, mean (SD)		5.3 (1.3)	4.1 (1.1)	.12	2.7 (1.6)	.03	2.0 (0.6)^+^	.02	—^g^
qDASH^i^ score, mean (SD)		30.6 (18.1)	15.5 (7.5)	.03	11.4 (9.5)	.02	9.8 (12.4)	.02	—^g^

^a^MCID: minimal clinically important difference. Computed using a distribution-based method using the standard error of measurement (SEM). SEM = SD of the baseline measurements * √1-reliability, where the reliability used was retrieved from the intraclass correlation coefficient, and MCID = SEM * 1.96 * √2.

^b^Internal rotation level.

^c^T12 vertebra.

^d^Waist (L3).

^e^Lumbosacral junction.

^f^Interscapular (T7).

^g^Not applicable.

^h^VAS: visual analogue scale.

^i^qDASH: Quick Disabilities of the Arm, Shoulder, and Hand questionnaire.

**Table 5:** Improvement in parameters compared with baseline and with time in the home-based exercise group.

Parameters		Baseline	1 month	*P* value	2 months	*P* value	3 months	*P* value	MCID^a^
**Abduction (°), mean (SD)**									
	Active	113.1 (20.3)	115.9 (26.4)	.35	127.9 (21.6)	.04	134.3 (21.8)	.051	5.99
	Passive	131.6 (17.1)	133.9 (15.9)	.40	135.3 (15.6)	.18	142.7 (18.2)	.13	4.63
**Flexion (°), mean (SD)**									
	Active	127.9 (11.0)	132.3 (15.1)	.13	138.0 (13.7)	.03	140.0 (11.2)	.03	6.78
	Passive	146.7 (7.3)	148.6 (9.5)	.35	152.7 (8.3)	.07	154.3 (8.1)	.13	7.14
**Extension (°), mean (SD)**									
	Active	54.1 (5.0)	55.7 (6.9)	.50	58.3 (4.6)	.26	56.0 (5.9)	.35	11.15
	Passive	63.1 (6.6)	63.3 (8.0)	.93	65.9 (6.4)	.50	68.3 (6.3)	.27	11.36
**External rotation (°), mean (SD)**									
	Active	49.0 (9.7)	50.9 (8.7)	.46	51.1 (8.4)	.45	53.1 (12.9)	.13	5.88
	Passive	58.0 (11.9)	58.7 (10.2)	.67	59.1 (10.2)	.40	60.9 (11.5)	.13	4.44
**Internal rotation (level)^b^, mean (SD)**									
	Active	1^c^, 2^d^, 4^e^	1^c^, 2^d^, 3^e^,1d	.56	1^c^, 2^d^, 4^e^	>.99	1^c^, 2^d^, 3^e^, 1^f^	.56	—^g^
	Passive	1^c^, 4^d^, 2^e^	1^c^, 3^d^, 3^e^	.56	2^c^, 3^d^, 2^e^	.66	3^c^, 2^d^, 2^e^	.41	—^g^
VAS^h^ score, mean (SD)		6.1 (1.8)	5.6 (1.7)	.46	4.1 (1.1)	.04	3.3 (1.1)	.02	—^g^
qDASH^i^ score, mean (SD)		23.3 (7.2)	24.4 (18.7)	.80	19.8 (12.1)	.35	19.1 (13.7)	.25	—^g^

^a^MCID: minimal clinically important difference. Computed using a distribution-based method using the standard error of measurement (SEM). SEM = SD of the baseline measurements * √1-reliability, where the reliability used was retrieved from the intraclass correlation coefficient, and MCID = SEM * 1.96 * √2.

^b^Internal rotation level.

^c^Interscapular (T7).

^d^T12 vertebra.

^e^Waist (L3).

^f^Lumbosacral junction.

^g^Not applicable.

^h^VAS: visual analogue scale.

^i^qDASH: Quick Disabilities of the Arm, Shoulder, and Hand questionnaire.

A comparison of the improvements in the motion sensor–assisted rehabilitation and home-based exercise groups over time revealed that, compared with patients in the home-based exercise group, those in the motion sensor–assisted rehabilitation group had significantly better pROM in shoulder abduction, flexion, extension, and external rotation; better aROM in shoulder extension and internal rotation; and a better qDASH score at the 3-month follow-up (Table 6). In addition, the changes in shoulder ROM in most of the directions at different follow-up time points from baseline were correlated with improvements in qDASH score, inferring that the motion sensor device is a reliable tool for evaluating treatment efficacy in patients with AC (Multimedia Appendix 4). Table 7 shows that, compared with patients in the home-based exercise group, those in the motion sensor–assisted rehabilitation group had a significantly higher patient-reported exercise completion rate.

**Table 6:** Generalized estimating equation analysis for improvements between groups with time (reference: home-based exercise group).

Dependent variables			1 month, beta (SE)		*P* value		2 months, beta (SE)		*P* value		3 months, beta (SE)		*P* value
**Abduction (^o^)**													
	Active	23.00 (10.49)		.03		17.00 (8.09)		.04		13.14 (9.83)		.19	
	Passive	23.71 (8.37)		.006		27.00 (5.49)		<.001		23.57 (8.38)		.007	
**Flexion (^o^)**													
	Active	6.29 (3.29)		.06		2.43 (6.00)		.69		9.00 (5.33)		.10	
	Passive	13.71 (2.78)		<.001		12.29 (6.10)		.049		16.14 (4.87)		.002	
**Extension (^o^)**													
	Active	4.71 (3.52)		.19		5.29 (3.86)		.18		15.29 (6.03)		.01	
	Passive	5.29 (3.57)		.14		11.57 (5.98)		.06		15.29 (5.72)		.01	
**External rotation (^o^)**													
	Active	12.14 (3.31)		<.001		10.14 (4.11)		.02		8.00 (4.50)		.08	
	Passive	5.14 (3.90)		.19		9.00 (3.12)		.006		12.14 (3.74)		.002	
**Internal rotation (level)^a^**													
	Active	1.00 (0.28)		<.001		1.14 (0.37)		.003		1.29 (0.40)		.002	
	Passive	0.57 (0.31)		.07		0.57 (0.41)		.17		0.43 (0.43)		.32	
VAS^b^ score			–0.64 (1.15)		.58		–0.57 (0.94)		.55		–0.43 (0.70)		.54
qDASH^c^			–16.19 (8.28)		.06		–15.70 (6.11)		.01		–16.60 (5.97)		.007

^a^Improvement at the internal rotation level.

^b^VAS: visual analogue scale.

^c^qDASH: Quick Disabilities of the Arm, Shoulder, and Hand questionnaire.

**Table 7:** Exercise completion rate reported by the participants and motion sensor device.

Time point	Motion sensor–assisted rehabilitation group		Home-based exercise group	*P* value^a^
	Recorded by motion sensor device	Reported by participants	Reported by participants	
1 month, n (%)	80.0 (21.2)	82.1 (19.1)	35.7 (18.1)	.007
2 months, n (%)	79.5 (23.2)	85.6 (19.8)	36.4 (19.1)	.007
3 months, n (%)	78.5 (26.9)	89.9 (5.5)	35.0 (17.6)	.002
Overall mean (SD)	79.3 (22.7)	86.0 (13.5)	35.7 (13.2)	.002

^a^Comparison of the participant-reported exercise completion rates between the motion sensor–assisted rehabilitation and home-based exercise groups.

## Discussion

### Principal Findings

This study revealed that wearable IMU-based sensors can measure shoulder ROM with acceptable reliability. When the wearable motion sensors were integrated with interactive mobile apps and telecommunication technology for the treatment of AC, patients could correctly perform daily home-based shoulder exercises under the remote supervision of a physician, as revealed by a 3-month ambulatory assessment. Consequently, compliance with the daily shoulder exercise regime was enhanced, thus improving the recovery of shoulder ROM and function among patients with shoulder AC using motion sensor device–assisted rehabilitation.

Measurement of shoulder ROM is an important component of related clinical assessments, providing information that is useful for guiding and evaluating interventions for patients with AC. Many methods can be used to measure ROM including a goniometer, 3-dimensional motion analysis (3DMA) systems, and IMU-based sensors. The goniometer is widely used but has variable reliability and tends to fail in assessing joint angles during dynamic movement [25]. The 3DMA system, which is regarded as the gold standard for evaluating lower limb kinematics [26], measures joint angles during dynamic movement with multiple degrees of freedom. However, the use of the system to measure shoulder ROM is technically challenging owing to the complexity of shoulder movement [27]. Moreover, the 3DMA system is typically immobile, difficult to access, and requires expertise to operate, preventing its general clinical use. Wearable IMU-based sensors are becoming popular and have the potential to measure the joint angles of upper limbs with acceptable reliability [28]. Studies have already revealed that wearable IMU-based sensors can track shoulder movements with high accuracy [29]. In the present study, shoulder ROM measurement by IMU sensors yielded good to excellent interrater reliability with reports from well-experienced physicians who were using goniometers; it can therefore provide reliable angular information that physicians can use to monitor the progress of shoulder rehabilitation and facilitate the design of upper limb exercises that promote the rehabilitation of patients with AC.

An important aspect of the motion sensor device used in this study is the integration of a mobile phone–based system. Mobile phone apps for medical and rehabilitation purposes that are adaptable and easily accessible are being intensively researched [29,30]. Such apps may help in the development of a platform for delivering self-management interventions, thus improving the delivery of health care and outcomes [28]. Evidence has also revealed that app-based exercise instructions and tools can help patients to monitor their training compliance and progress with high acceptance and usability [29]. In this study, the mobile apps with the motion sensor device system provided an informative patient interface with comprehensible exercise instructions and simple progress monitoring; they support self-managed health care anywhere through the monitoring of rehabilitation exercise performance. Wireless telecommunication is used to synchronize with the Doctor App, send information regarding each patient’s progress with home-based exercises, enable physicians to remotely supervise rehabilitation, and provide instant feedback to patients. Studies have established that the delivery of simple text message reminders to mobile phones increases the compliance of AC patients to a shoulder exercise regimen [31]. With the assistance of a mobile app that is used in conjunction with a motion sensor device for shoulder rehabilitation, physiotherapists or physicians can not only send text messages to remind patients to perform their daily exercises but also assign personal home-based exercises based on their training performance, thus increasing their motivation.

Overall, the positive results justify further work on motion sensor devices for treating AC. Further investigations of the usability of mobile apps and the development of auto-adjusted rehabilitation programs that are based on a user’s personal performance are warranted before well-controlled research is conducted with large patient cohorts to demonstrate the viability of motion sensor devices in real-world environments. The motion sensor device–assisted rehabilitation model in this study can be applied in conjunction with telerehabilitation. Telerehabilitation is the provision of rehabilitation services at a distance; it can be image-based or sensor-based and can be delivered using virtual environments or virtual reality [32]. A motion sensor device can support image-based and sensor-based telerehabilitation using an activity recognition model, an interactive 3D avatar in mobile phone apps, and a wireless telecommunicated network, supporting medical treatment for patients with AC. The future use of motion sensor devices in telerehabilitation for various muscle skeletal disorders is expected after its development reaches maturation.

### Limitations

The main limitations of this study were the relatively small number of patients, short follow-up period, and consequent lack of information on the long-term effects of the intervention. The allocation of patients was not randomized, owing to their varying degrees of familiarity with mobile apps and motion sensor devices. Studies have found that new rehabilitation technology may be unlikely to be accepted by many elderly patients [33]. In this study, the targeted patients with AC were 40-70 years old. Within this population, younger and highly educated patients are generally accepting of motion sensor device–assisted rehabilitation and so were likely to be allocated to the motion sensor–assisted rehabilitation group. Researchers of future studies should consider a training program for motion sensor device usage before enrollment to prevent any potential selection bias. Finally, although this study revealed that the IMU-based sensors that were used to measure shoulder ROM were as reliable as goniometers used by two well-experienced physicians, the true accuracy of IMU-based sensors for shoulder ROM measurement is still unclear. Evidence has confirmed that the reliability of shoulder and elbow ROM measurement using the goniometer varies (interrater ICC, 0.36-0.91) [25]. Further assessment may be required to compare IMU-based sensors and the 3DMA system in the measurement of shoulder ROM with regard to accuracy and reliability.

### Conclusions

Wearable IMU-based sensors can reliably record the angular motion of shoulders. Motion sensor device–assisted home-based rehabilitation can increase patient compliance with a daily self-exercise regime and facilitate the early stage of functional recovery for patients with shoulder AC. Collectively, motion sensor device–assisted home-based rehabilitation is a useful treatment model for AC with telerehabilitation to overcome obstacles to physiotherapy at home; it provides comprehensive and easily accessible exercise instructions, increases compliance, and enhances exercise correctness through progress monitoring.

## Appendix

Multimedia Appendix 1 BoostFix Quick Guide.

Multimedia Appendix 2 Accuracy of Angular Measurement obtained with Single-Sensor and Dual-Sensor Systems.

Multimedia Appendix 3 Home-based Exercise Protocol for Shoulder Adhesive Capsulitis.

Multimedia Appendix 4 Correlation between changes in qDASH score and shoulder ROM in the different directions at different time points of follow-up from baseline.

## Abbreviations

AC: adhesive capsulitis
aROM: active range of motion
3D: 3-dimensional
3DMA: 3-dimensional motion analysis
ICC: intraclass correlation coefficient
IMU: inertial measurement unit
MCID: minimal clinically important difference
pROM: passive range of motion
qDASH: Quick Disabilities of the Arm, Shoulder, and Hand
ROM: range of motion
SEM: standard error of the measurement
VAS: visual analogue scale
