# Supplementary material for: Wearable Motion Sensor Device to Facilitate Rehabilitation in Patients With Shoulder Adhesive Capsulitis: Pilot Study to Assess Feasibility
Source: J Med Internet Res. 2020 Jul 23;22(7):e17032. doi: 10.2196/17032 (PMC7413285; doi:10.2196/17032)
Supplement: Multimedia Appendix 1 [file jmir_v22i7e17032_app1.docx]

**BoostFix Quick Guide**

Version 1.0

[1 Descriptions of intended device users, uses, use environments, and training 3](#_Toc33632687)

[1.1 Device description 3](#_Toc33632688)

[1.2 User population 3](#_Toc33632689)

[1.3 Intended Use 4](#_Toc33632690)

[1.3.1 Prescribing exercises 4](#_Toc33632691)

[1.3.2 Determining akinesia through real-time recordings 4](#_Toc33632692)

[1.3.3 Monitoring range of motion in healthy users 5](#_Toc33632693)

[1.4 Device use environments 5](#_Toc33632694)

[1.4.1 Space required 5](#_Toc33632695)

[1.4.2 Device Maintenance 5](#_Toc33632696)

[1.4.3 Internet connection and security required 6](#_Toc33632697)

[1.5 User training 7](#_Toc33632698)

[2 Description of device user interface 8](#_Toc33632699)

[2.1 Overview 8](#_Toc33632700)

[2.2 Device packaging 8](#_Toc33632701)

[2.2.1 Standard Packages 8](#_Toc33632702)

[2.3 QHP’s login and functions 9](#_Toc33632703)

[2.3.1 QHP’s login 9](#_Toc33632704)

[2.3.2 Settings 10](#_Toc33632705)

[2.3.3 Patients 11](#_Toc33632706)

[2.4 Patient’s log-in and functions 12](#_Toc33632707)

[2.4.1 Patients 14](#_Toc33632708)

[2.4.2 Device setup 14](#_Toc33632709)

[2.4.3 Prescribed exercises 18](#_Toc33632710)

[2.4.4 Real-time recordings 19](#_Toc33632711)

[2.5 Built-in data analysis 22](#_Toc33632712)

[2.5.1 QHP data review 22](#_Toc33632713)

[2.5.2 Patient data review 22](#_Toc33632714)

1. Descriptions of intended device users, uses, use environments, and training
   1. Device description

BoostFix , developed by Compal Electronics, Inc., is a medical device comprising 1) sensors for recording angular motion of a user/patient; 2) an accessory mobile application, “Patient app”, to be utilized by the user/patient; and 3) another accessory mobile application, “Doctor app”, to be used by a qualified healthcare professional (QHP). The intended use of this device is to create a platform whereby rehabilitative and diagnostic movements can be quantified and tracked, thus enhancing the quality of treatment for potential users.

- 1. User population

There are two primary subsets of users for this medical device. The first subset of users includes qualified healthcare professionals (QHPs), especially those in the fields of Physical Medicine and Rehabilitation, Occupational Therapy, Physical Therapy, and Health and Fitness. QHPs must be trained on both accessory mobile applications and on the sensor device. QHPs will primarily use the “Doctor App” to create patient accounts, prescribe exercise movements via the app, and monitor patient-recorded data. QHPs are also responsible for operating the sensors in a clinical environment. QHPs will be responsible for training their respective patients on appropriate use of the sensors and operating the “Patient App”.

According to the evaluation of BoostFix, QHP users should have the following characteristics:

- - - General knowledge of both accessory mobile applications, Wi-Fi, and Bluetooth
    - Ability to learn a new mobile application
    - Ability to learn how to operate a medical device
    - Access to an iPad with minimum requirements (Appendix x) and Wi-Fi in the working environment

The second subset of users includes patients who have been registered by a QHP for recording their movements. These users are responsible for operating the device sensors and the “Patient App”. These users will operate the device either “in clinic” or “at home”.

According to the evaluation of BoostFix, patient users should have the following characteristics:

- - - Physical dexterity to don sensors independently; otherwise, with the aid of another individual
    - General knowledge of mobile applications, Wi-Fi, and Bluetooth
    - Ability to learn a new mobile application
    - Ability to learn how to operate a medical device
    - Ability to remember or reference a 10-digit username and its unique password
    - Access to an iPhone with minimum requirements and Wi-Fi for at-home operation


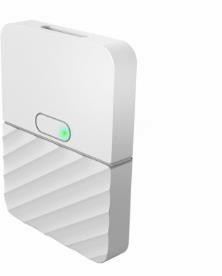

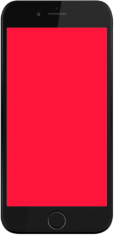

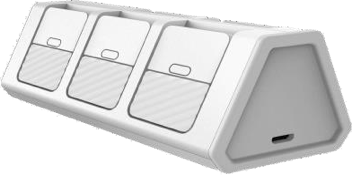

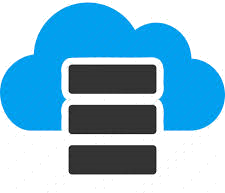

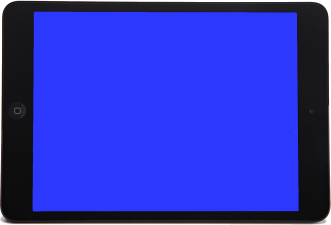

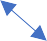


**BoostFix**

**sensor**

**BoostFix**

**cradle**

Hz) or Bluetooth data

transmission (10 Hz)

Bluetooth

Sensor connection

Wi-Fi data transmission (30

**Patient iOS**

**device**

**iOS QHP’s**

**device**

Wi-Fi sync

Wi-Fi sync

**Secure**

**cloud**

Figure 1. Information relay between device and secure cloud

- 1. Intended Use
     1. Prescribing exercises

One intended use of this device is to provide a platform whereby QHPs can prescribe exercises for patients to perform at home or in clinic. A general overview of the information relay between the device and its data upload is presented in Figure 1. A QHP’s personal account is set up by support team (Support team will set up accounts for the US market). This private account allows a QHP to then create users for each of his/her patients that will use a device. When the QHP creates a new patient account, both personal (i.e., age, phone number, etc.) and medical information (i.e., diagnosis) of the patient user is entered. Then, specific relevant exercises are selected by the QHP and added to the patient’s “prescribed exercises” tab. Each of these exercises can be tailored in terms of frequency, goal angle, tolerance, and duration.

- - - Frequency: number of times to repeat an exercise in a given period
    - Goal: Target angle to be achieved
    - Tolerance: ± angle range allowed around the goal angle
    - Duration: seconds of rest before next activity

After the patient user has performed his/her prescribed exercises using the “Patient App”, recorded measurements are uploaded to a HIPAA-compliant cloud. This cloud syncs the data with the QHP’s account, so that the QHP is able to see the recorded movements and extracted data. The prescription of exercises via the apps allows QHPs to track progression of range of motion (ROM) and motion fluidity of their patients over an extended time period.

- - 1. Determining akinesia through real-time recordings

Another feature of this device is the real-time recording of movements involved in activities of daily living (ADL), such as gait and reach/grasp. The use of an avatar playback on the apps with displayed angle measurements allows the QHP to observe the user’s movements and detect compensatory motions or areas with decreased ROM during natural movements.

For such movements, an area with adequate space (either at home or in clinic) to perform the movement is needed. All prescribed movements must not exceed the Bluetooth detection range.

- - 1. Monitoring range of motion in healthy users

This device may also be used with patient users that have normal ROM for the purposes of 1) tracking ROM to detect changes later in time; and 2) creating a reference dataset for future use (i.e., post-surgery) or developing new therapies for future patient users.

- 1. Device use environments
     1. Space required

BoostFix is intended for use in both clinical and non-clinical (e.g., residential fitness facilities) settings. The environment requires a clear area where a user can perform movements such as bending at the waist and lifting legs. In some scenarios, accessory tools may be required, such as a chair to sit on or a wall/bar for maintaining balance. For real-time recordings, the required space may be larger, depending on the exercise prescribed.

- - 1. Device Maintenance

The BoostFix cradle has an average battery life of 24 hours and the sensors have an average battery life of 7 hours. The cradle can be charged by plugging it into an AC adapter provided in the packaging. A micro-USB connection connects the cradle to an AC adapter provided in the packaging. This adapter needs to be plugged into a main power source and the cradle must be turned on for the device to charge. A blinking light, alternating between blue and green (emitted from the Bluetooth LED) indicates that the cradle is charging. Once charging is complete, a solid green LED light will show (Figure 2a). The sensors have a charging pin (Figure 2b) that should make contact with the pins on the cradle (Figure 2c). To charge, the sensor must be turned on. A charging status is indicated by an alternating blue and green light. A solid green light indicates charging complete. iPhone and iPad running the apps should neither have a battery charge below 20% nor in “Battery Save” mode. Otherwise, there will be problems with data collection and upload.


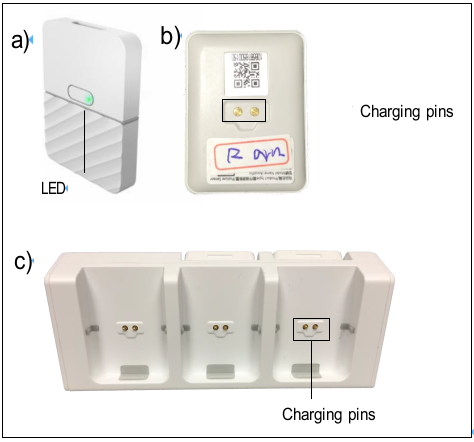


Figure 2. Information relay between device and secure cloud

While the sensors provided with each cradle can also be used with other cradles, the internal device identification number enables matching sensors with their original cradles. If a clinical room contains multiple cradles, the user may use colored stickers to group sensors with cradles. Additionally, numbering the sensors may facilitate setup. For example, one cradle may be dedicated to upper limb tracking while another cradle may be dedicated to lower limb tracking. Numbered sensors allow the user to place sensors in pre-determined locations. However, the device allows reconfiguring sensors and locations via functions in the app, whenever needed.

- - 1. Internet connection and security required

For both “Doctor App” and “Patient App” to work, the tablet/phone on which the apps are installed must be connected to a secure Wi-Fi network and have Bluetooth turned on. The Wi-Fi connection should have password-protected access limited to only essential personnel, if in clinic; and to a private network, if at home. Without such security ensured, private medical information and data may be exposed to unauthorized access. In order to connect the motion sensor device to the Wi-Fi network, the user must know the password. Compal has ensured that all uploaded data to servers or clouds are HIPAA-compliant. The structure of the connections involved in the usage of this device are presented in Figure 1 and previously described. Compal has performed testing to ensure no interruption from other devices communicating via Bluetooth in the vicinity.


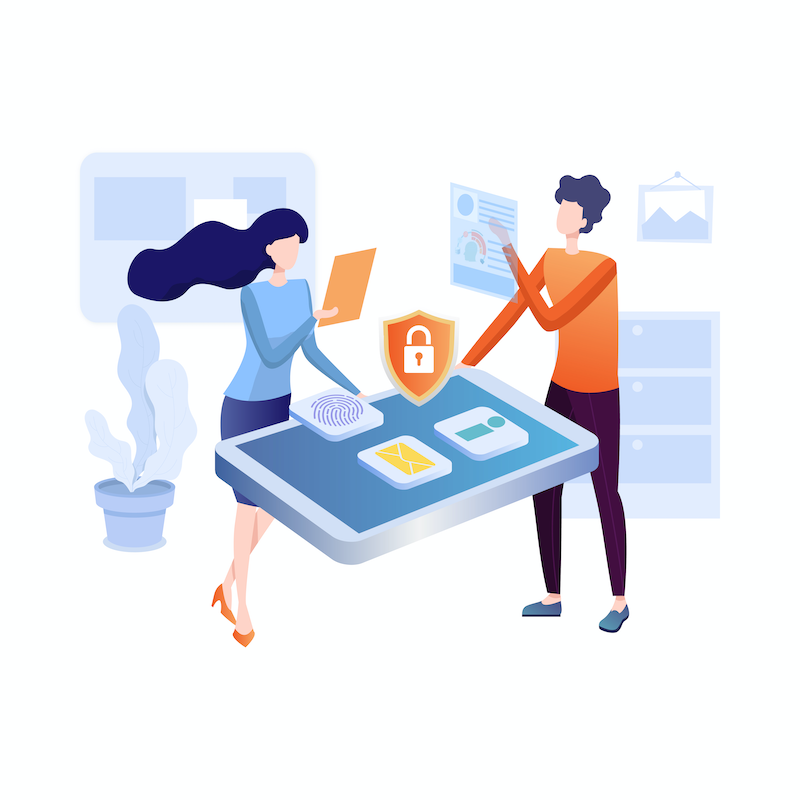


Figur.3 Internet security support

- 1. User training

Two different manuals for user training will be developed. These manuals will be made available in printed form with the device and also available via download (link to be provided with printed version).

For a first-time user, a trained representative will make an on-site visit with the lead QHP. This representative will train the QHP, ensure proper Wi-Fi and Bluetooth operation, and validate upload of data to the approved cloud server. The representative will also provide the QHP with his/her login information for the account.

The training for a QHP involves:

- - - General iPhone and iPad operations (Wi-Fi connect, Bluetooth connect, app download/update procedures)
    - Logging into QHP’s account
    - Adding new users (patients) and their personalized exercises
    - Adding custom-designed exercises
    - Syncing app with cloud to see updated patient data
    - Operating the device
      - Turning on/off and charging the cradle and sensors
      - Verifying Wi-Fi and Bluetooth connection
      - Attaching sensors to a user (Velcro straps, body placements)
      - Registering a sensor to a specific position on the app (using avatar); releasing a sensor; checking battery level of sensor; checking sensor position
      - Performing measurements and real-time functions
    - Operating the “Patient App” and training a potential patient user

The training (given by a QHP) for a patient user involves:

- - - General iPhone and iPad operations (Wi-Fi connect, Bluetooth connect)
    - First-time login password change
    - Operating the device
      - Turning on/off and charging the cradle and sensors
      - Verifying Wi-Fi and Bluetooth connection
      - Attaching sensors (Velcro straps, body placements)
      - Registering a sensor to a specific position on the app (using avatar); releasing a sensor; checking battery level of sensor; checking sensor position
      - Performing measurements and real-time functions

1. Description of device user interface
   1. Overview

A general overview of steps involved in device usage is provided and can be used for reference throughout the remainder of this section. During these various steps, the two different users (QHP, blue; Patient, orange) will interact with different components of the device. In an initial meeting, a Compal representative (green) will be required to set up the QHP’s account and train him/her on proper device usage.

- 1. Device packaging
     1. Standard Packages

All devices and component in Figure 4.


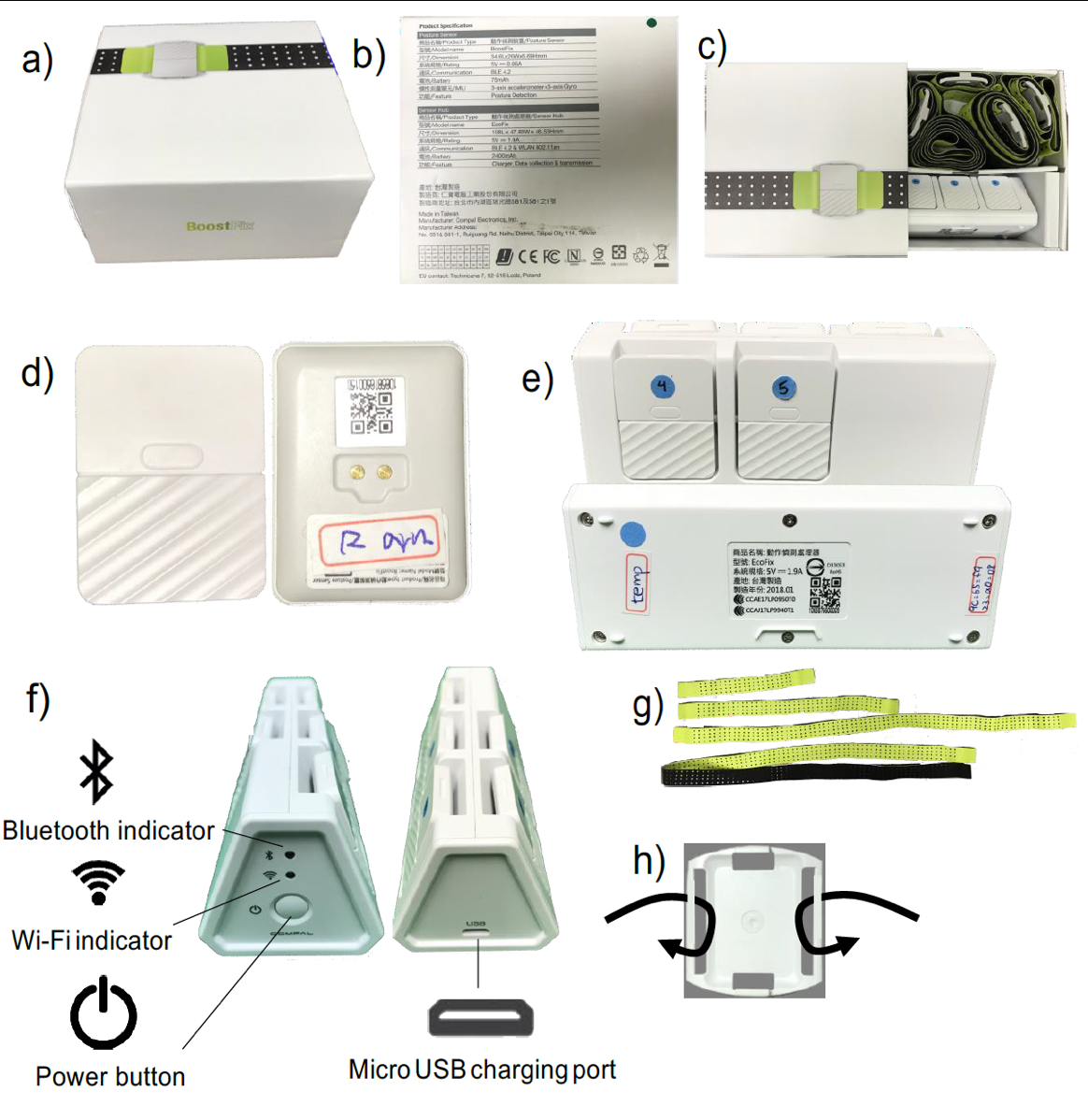


Figure 4. Device packaging and component

- 1. QHP’s login and functions
     1. QHP’s login

In order to use this device, the QHP must have an iPad. The “Doctor App” will be made available from the Android and iOS app stores. The QHP will be provided with a unique user account number and will be able to create a unique password during training with the Compal representative. Any subsequent password changes can be made by contacting Compal. The “Doctor App” and the “Patient App” can be distinguished by their respective colors (icon for the “Doctor App” contains a blue circle while the icon for the “Patient App” contains a red circle — see Figure 5). Upon clicking the “Doctor App” icon, the user will be taken to a login page (Figure 6a) and is required to enter an account number and password to gain access to his/her account.


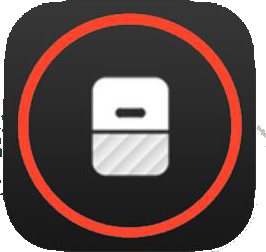

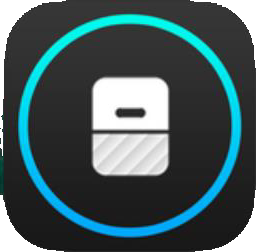


a) b)

Figure 5. Icon for “Doctor App” (a) and “Patient App” (b).


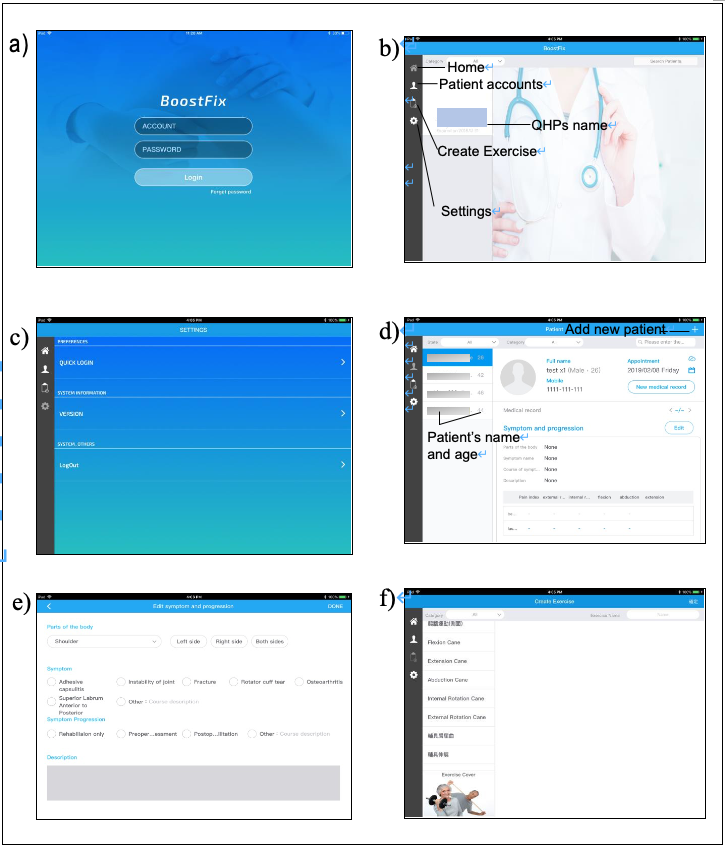


Figure 6. Screenshots of QHP mobile app.

- - 1. Settings

After logging in, the user is presented with the home screen (Figure 6b). From here, the QHP can navigate to multiple pages (tab options are located on the left side in a dark gray banner). The ‘Settings’ page (Figure 6c) allows the QHP to set up a ‘Quick Login’ which uses a fingerprint to access the account. Under ‘System Information’, the application version number and last update date can be found. Finally, a ‘Log Out’ option is provided for quitting the QHP account and re-entering the ‘Log-in’ screen.


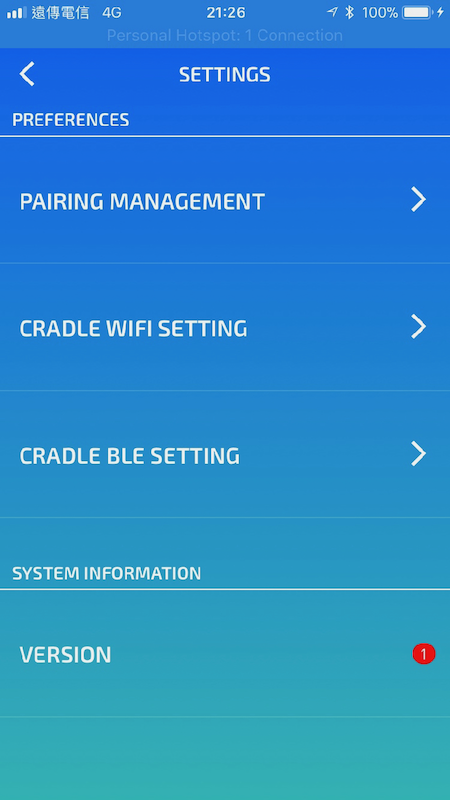


Figure.7 Setting menu

- - 1. Patients

Upon clicking the ‘Patient’ tab, the user is presented with a list of all patients that have been registered with account (Figure 6d). By default, the first patient’s information is selected, but the user can click ~~on~~ any other patient to see his/her account. The patient’s name, gender, and age can be seen from the patient list. After clicking an individual account, the patient’s full name, account number, next appointment date and medical record can be seen. Multiple medical records can be added to a patient’s account. Each medical record contains information about a single diagnosis. For a particular diagnosis, the part of the body (body part and side of body (left, right, or both)), symptoms, symptom progression, and description must be entered. The ‘Description’ tab allows the QHP user to add notes for the patient. Below the medical records, the ‘Exercise Prescription’ section holds all exercises prescribed to the patient. The QHP can add a new prescription by clicking ‘New’. The app has a selection of built-in exercises.

In the top right corner of this page, a plus sign (+) is available. The QHP can click ‘+’ to add a new patient.

- 1. Patient’s log-in and functions

The “Patient App” can be downloaded from the Apple iTunes store. The symbol for this version of the app is shown in Figure 5b. The first time a registered patient user logs into the app (Figure 8a), he/she will use the 10-digit code that was used during the doctor’s initial registration as the login and password. Then, the patient user will be prompted to change the password and login again.


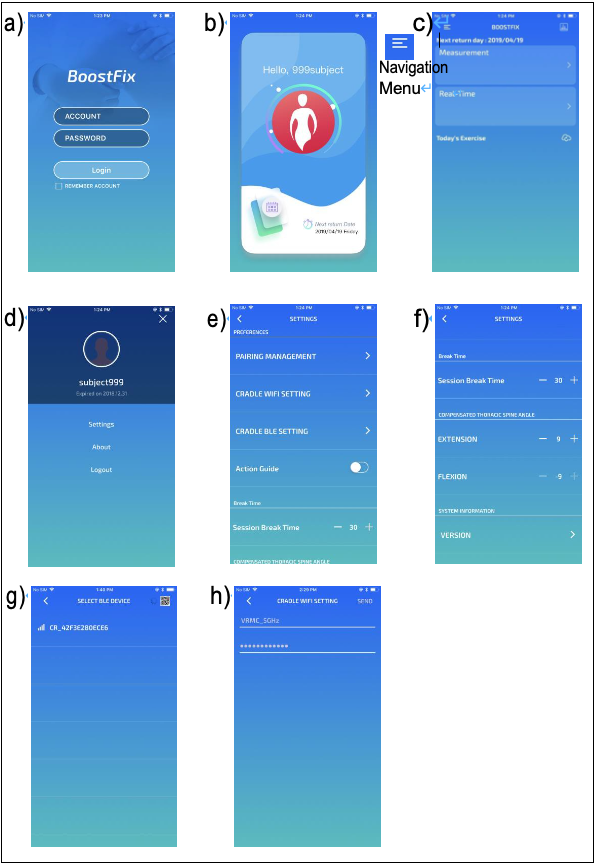


Figure 8. Screenshots of patient mobile app.

- - 1. Patients

Upon logging in, the patient will be taken to his/her home screen with either a blue or red circular button in the middle. The next appointment date is shown on the bottom right of the screen (Figure 8b). Upon clicking the circular button, the user will be taken to their exercise prescription page (Figure 8c). In the top left corner, a menu button (Figure 8c) is available. The user can use the navigation menu (Figure 8d) to navigate to the ‘Settings’ tab on the app. Under ‘Settings’ (Figure 8e), there are a few steps that need to be completed in order to set up the device and are described below. Other available options in this ‘Settings’ tab include turning ‘Action guide’ on and off, adjusting the seconds in the ‘Session Break Time’, and adjusting the compensatory angle. The compensatory angle refers to the angle that the torso sensor may sit ~~at~~ on the particular patient. An average angle of 15° has been determined from previous testing and is used as the default but can be adjusted after measuring the patient’s torso angle with a goniometer (not provided).

- - 1. Device setup

In order to set up the device for motion recording, the cradle should be turned on (indicated by a blinking blue LED light). First, the user should select the ‘Cradle Setting’ tab to connect to a device. The device ID of all available cradles within range will appear on screen (Figure 8g). To connect, the user simply taps on that particular cradle ID. Alternatively, the user can use the QR code scanner (by clicking the button in the top right corner) to scan the QR code of the cradle, which is located on the bottom of the device. This option may be more helpful when multiple cradles are available and the user is unsure of the appropriate cradle ID. If BLE connection is successful, a check mark will appear by the cradle ID number and the cradle BLE LED will become solid blue, rather than blinking blue.

Next, the user should connect to the Wi-Fi network by selecting the ‘Cradle Wi-Fi Setting’ tab under the ‘Settings’ page. The name of the Wi-Fi network that the phone is connected to will populate the first entry (Figure 8h). The user should enter the password for this particular network. Then, the user clicks the ‘Send’ button in the top right corner to establish the connection. If the credentials are correct, a notice will appear saying, ‘Setting Connection Success.’ Additionally, the Wi-Fi LED on the cradle will become solid blue. If not, an error notice will appear, and the user will be prompted to re-enter the password.

The user can then move on to setting up the sensors. When the sensors are first taken out of the cradle, they should be placed on a flat surface and turned on. To turn on the sensor, the user must hold down on the button (located on the anterior of the sensor) until two quick vibrations are felt. The sensors should be kept in the flat position for ~10 seconds. After all the sensors have been turned on, they can be paired to specific locations using the ‘Pairing Management’ tab in the ‘Settings’ menu. This takes the user to the ‘Pairing’ page which displays cradle battery life and sensor pairing locations (Figure 9a). If the sensor has previously been paired to a location, this location will be registered with a check mark (Figure 9a). In order to release this sensor from its particular setting, the user can press the ‘Release’ button. Alternatively, to check which sensor is paired with a position, the user can press on the yellow check mark, which will cause the respective sensor to vibrate three times. Possible sensor locations are:


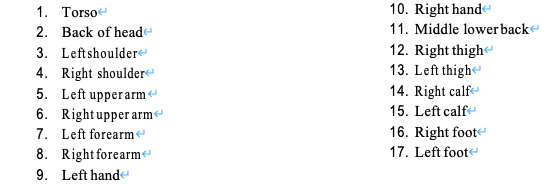


Figure 10 shows correct positioning of all sensors, according to the default calibration position (Figure 11a)


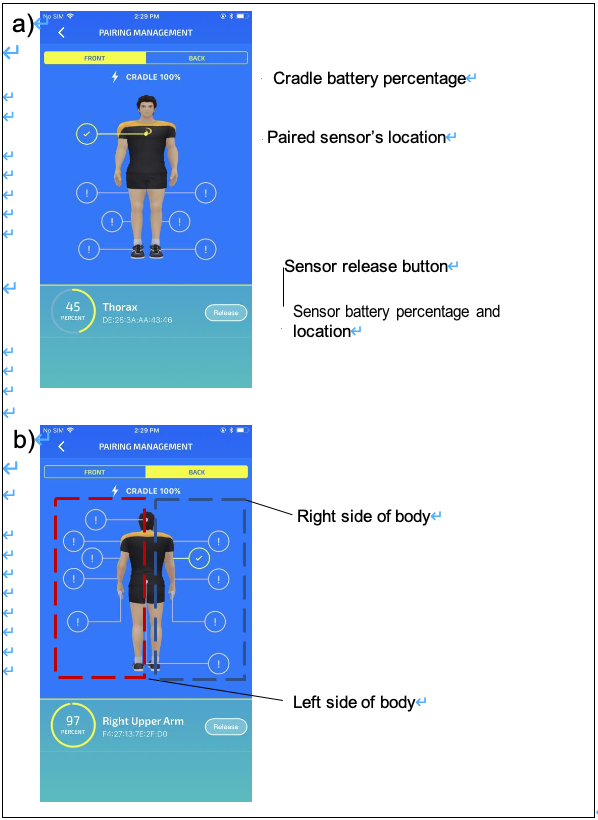


Figure 9. Screenshots of patient mobile app.


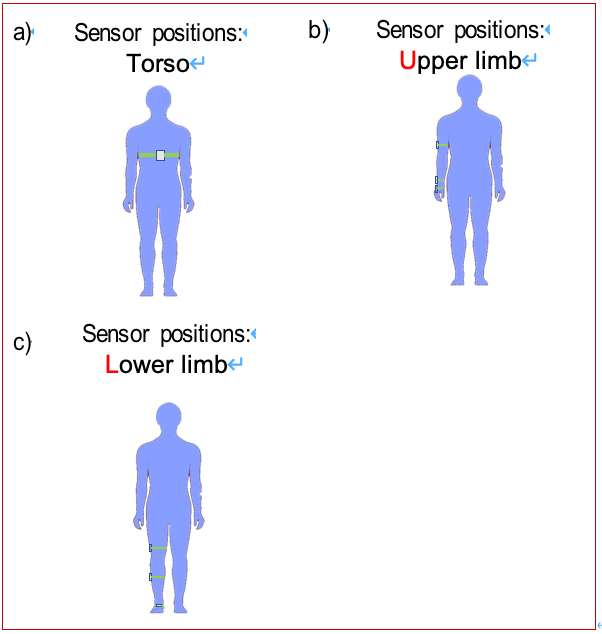


Figure 10. Sensor locations


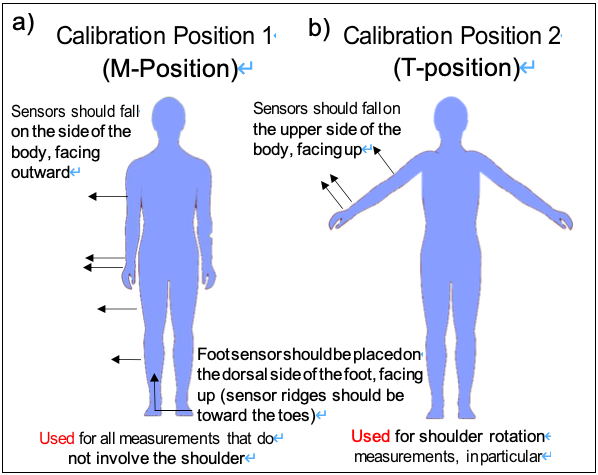


Figure 11. Calibration positions

- - 1. Prescribed exercises

The user has two options when recording his/her motions. In the patient’s home page, a tab for ‘Measurements’ and a tab for ‘Real Time’ are provided. Under ‘Measurements’, the user can select shoulder or knee to perform and record pre-uploaded exercises. Alternatively, the user can select exercises under ‘Today’s Exercises’ to access those that have been prescribed by his/her QHP. If the user selects ‘Measurements → Shoulder’ (Figure 12 a, b), he/she will be taken to all pre-uploaded exercises available for the shoulder. These include shoulder flexion, extension, internal rotation, external rotation, etc. (Figure 12c). Once an exercise is selected, results of the previous recording will be shown. The avatar animation will show how the exercise should be performed. Once the user is ready, he/she can press the ‘Start’ button, after which the calibration position needs to be performed (Figure 12d). Then the user may perform the movement after the countdown has finished. During this time, the current angle of the target joint is shown above the avatar (Figure 12e). Once the app’s internal data analysis system detects a stable position, the user will be asked to hold the position for an additional seven seconds. Once completed, the user will be told to return to the original position. Additionally, the user will be asked if he/she was assisted with the measurement. The user can click ~~on~~ ‘Yes’ or ‘No’ and ~~then~~ may continue to repeat the exercise or choose another exercise.


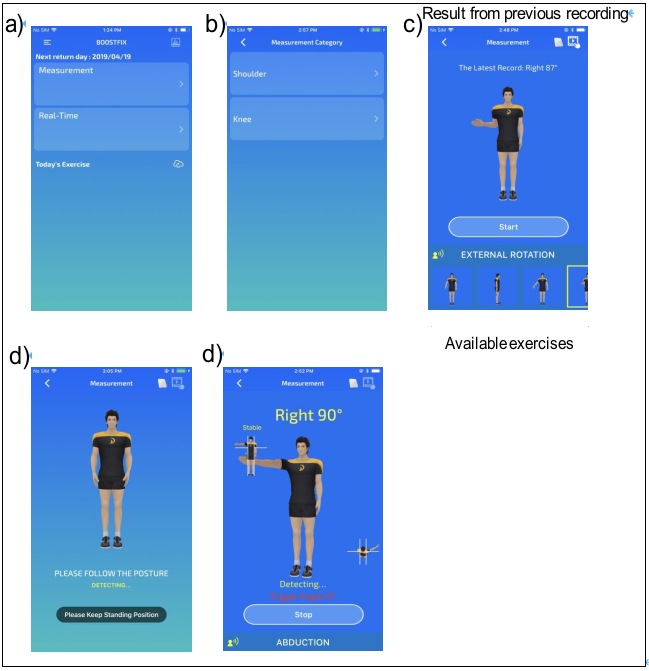


Figure 12. Screenshots of patient mobile app—performing a prescribed exercise

- - 1. Real-time recordings

In ‘Real-Time’, the user can record his/her movements while performing free (without the guidance of an avatar) movements. Upon clicking ‘Real-Time,’ the user is asked to stand in a calibration position (Figure 13 a and b). Here, the user will usually stand straight with their arms down by their sides. Upon holding the position for 3 seconds (counted down on the app), the user will be taken to a screen that shows the avatar performing the same movements as the user in real time. The user can rotate the screen by sliding a single finger on the avatar and can zoom in and out using two fingers. At the top of this screen (Figure 13c), the option for ‘Next Mode’ is available. Clicking this button takes the user to different perspectives of the avatar in real time. In the first mode (Figure 13c), only the avatar and no measurements are shown. In the second mode (Figure 13d), measurements related to right and left shoulder function are shown. In the third mode (Figure 13e), measurements from all body parts are shown. In the fourth mode (Figure 13f), the user has the option to alter parts of his/her recording with record, replay, and cut options. In all these modes, an option to record the movements is provided in the bottom right corner with a ‘Record’ button. Upon clicking the button, data will start recording. The ‘Record’ button then changes to a ‘Stop’ button. Upon clicking the button, a dialogue box will pop up, asking the user if he/she is sure about stopping the recording (Figure 13g). Upon clicking ‘Yes’, the recording will stop. To access all recordings, the user needs to click ~~on~~ the ‘Play’ button in the top right corner (Figure 13g). This will take the user to the list of all recorded movements (Figure 13h). Each of these recordings can be played back by first choosing an item, followed by clicking ~~on~~ the ‘Play’ button or ‘Real-time comparison’ button (Figure 13i). In the ‘Real-time comparison’ mode, the user can continue to move while the original recording on the avatar was being overlaid (Figure 13j).


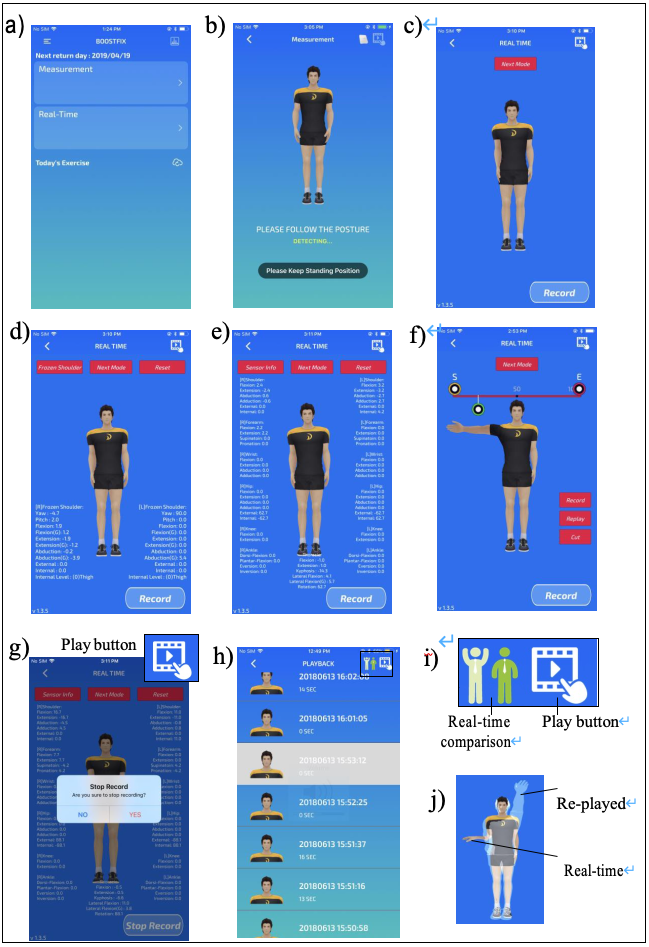


Figure 13. Screenshots of the patient mobile app—Real Time mode

- 1. Built-in data analysis
     1. QHP data review

All patient-recorded data are uploaded to a secure cloud, which are then synced with the doctor’s account. The ‘Cloud’ button located in the “Doctor App” allows the QHP to resync if necessary. Internal data analysis of recordings allows automatic measurement of the stable angles that users are able to reach. With date- and time - stamps, the QHP can track the ROM by day, week, and month (Figure 14a).


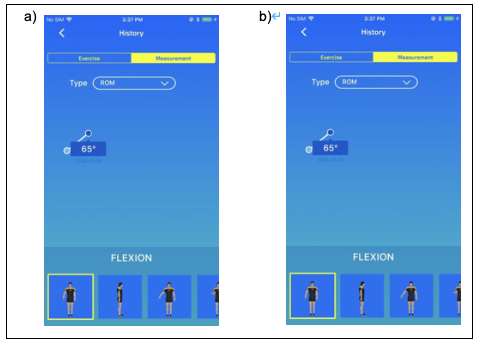


Figure 14. Internal data analysis of measurements available to QHP(a) and patient (b).

- - 1. Patient data review

A ‘History’ button is located in the top right corner of the patient’s home screen. Under this option,

information from previous exercises and measurements can be tracked by date (Figure 14b)
