# Supplementary material for: Wearable Motion Sensor Device to Facilitate Rehabilitation in Patients With Shoulder Adhesive Capsulitis: Pilot Study to Assess Feasibility
Source: J Med Internet Res. 2020 Jul 23;22(7):e17032. doi: 10.2196/17032 (PMC7413285; doi:10.2196/17032)
Supplement: Multimedia Appendix 2 [file jmir_v22i7e17032_app2.docx]

**Supplementary Report**

**Accuracy of Angular Measurement obtained with Single-Sensor and Dual-Sensor Systems**

1. **Introduction:**

BoostFix is a real-time signal acquisition device with sensors for inertial measurements. The sensors comprise six-axis microelectromechanical systems with accelerometers and gyroscopes, and collect information on the angular motion of the shoulder of interest.

A single-sensor system can offer information on the angular deviation (or rotation angle) on the x-, y-, and z-axes in space; while the dual-sensor system can shed light on the relative angular change for each sensor, computing the range of motion on the simulated joint movement. The purpose of this experiment is to verify the accuracy of the angular measurements obtained with the Boostfix sensors, which are designed to meet the specification of angular measurement error of less than ± 1°.

1. **Apparatus:**

###### Boostfix Sensor DVT sample set (v0.1.0)

Sensors, numbered: S1, S2, S3, S4 (FTP_43.0, FTP_55.10)

###### Boostfix cradle DVT sample set (v0.2.0)

###### Hardware: CEX01 (6-axis)

| Motion Sensor | InvenSense MPU-6500 |
| --- | --- |
| SoC | NORDIC nRF52832 |
| CPU | ARM® Cortex™-M4F |

###### One set of goniometer

###### Testing instrument description:

Producer: Baiju Ruler Co., Ltd.

Product name: 0.1° indexing goniometer

Product number: SP-01-500A

Production serial number: EB-1506

Brand name: E-Base

Measurement specifications: 0°-180°

Minimum reading: 0.1°

Error tolerance: +/- 0.2°

###### Data retrieval using Android phone (ZenfoneAR, Android 7.0)

###### Sensor angle verification APP (v0.1)

1. **Testing Goal**

### To verify the accuracy of angular deviation measured by a single sensor on the x/y/z-axis

### To simulate the shoulder joint movement with two sensors on a goniometer and verify the accuracy of angular measurements

1. **Testing step**
   1. **Single-sensor angular measurement**
      1.
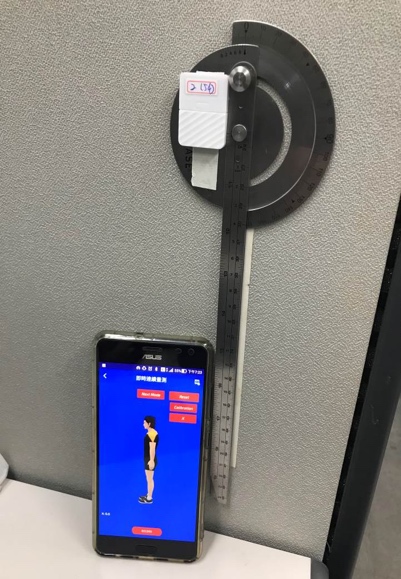
A sensor S1 was fixed on the moving arm of a goniometer which was located on the wall and calibrated on 0°. (Fig. 1)

**Figure 1 Single-sensor measurement approach**

- - 1. The angle of the goniometer was increased from 0° to 180° in stages of 30° each. The angular measurements obtained by the sensor and the goniometer were recorded. (Fig. 2)**
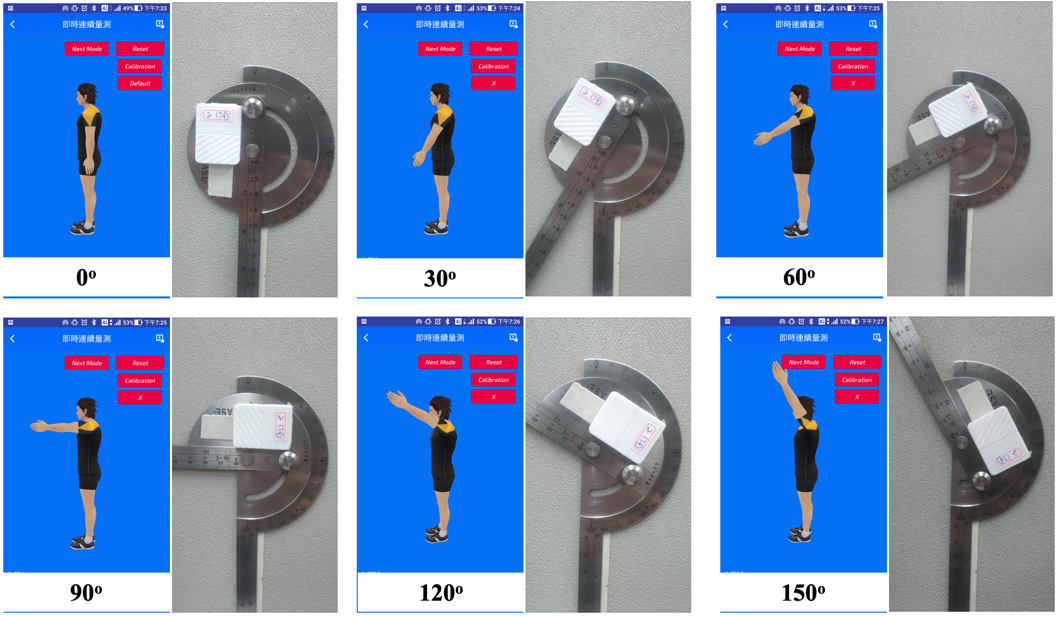
**

**Figure 2 Single-sensor angular measurements on different angles**

- - 1. Upon reaching 180°, the angle of the goniometer was decreased from 180° to 0° in stages of 30° each. The angular measurements obtained by the sensor and the goniometer were recorded.
    2. Sensor S1 was rotated or flipped and then fixed on the moving arm of the goniometer for recording angular changes on different axes (x/y/z- axis). (Fig. 3) Repeat Step 1 to Step 3.


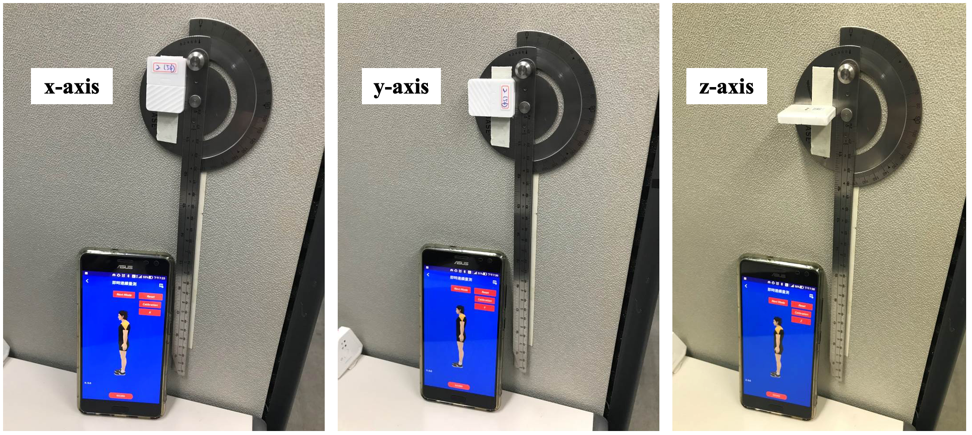


**Figure 3 Calibration of sensors on different axes**

- - 1. Sensor S1 was replaced with sensors S2 to S4. Sensors S1 and S2 tested the x-axis; sensors S2 and S3 tested the y-axis; and sensors S3 and S4 tested the z-axis
  1. **Dual-sensor angular measurement on a simulated shoulder movement**
     1.
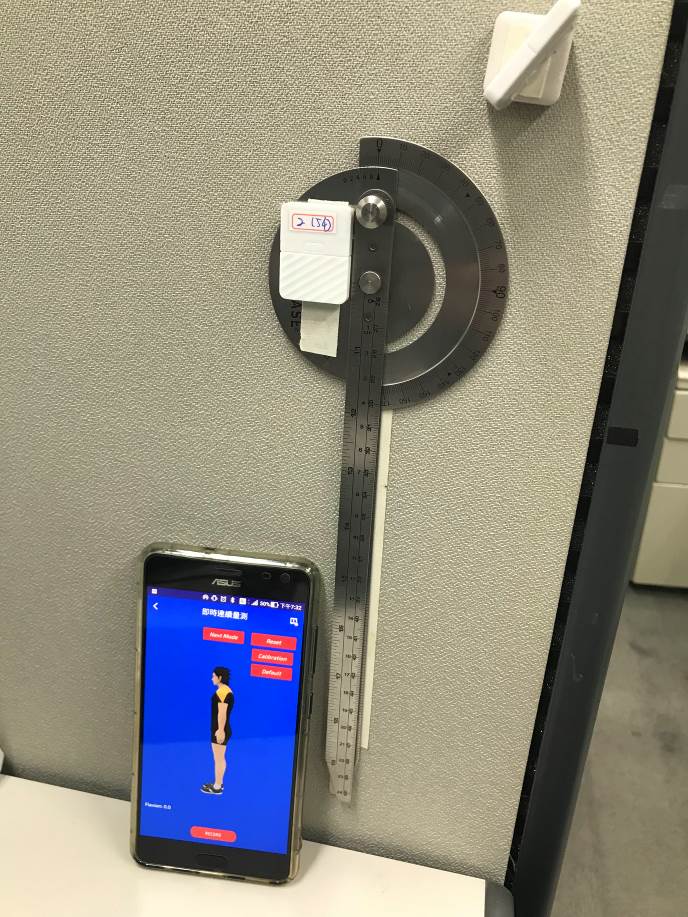
To simulate the scenario of the participant wearing two sensors, sensor S1 was fixed on the wall at a tilt angle of 40° to simulate the sensor worn on the sternum of the participant. (Fig. 4)

**Figure 4 Dual-sensor measurement to simulate shoulder movement**

- - 1. Another sensor S2, which simulated the sensor worn on the upper arm of the participant, was fixed on the moving arm of a goniometer which was located on the wall and calibrated on 0°. (Fig. 4)
    2. The angle of the goniometer was increased from 0° to 180° in stages of 10° each. The angular measurements obtained by the sensor and the goniometer were recorded. (Fig. 5)


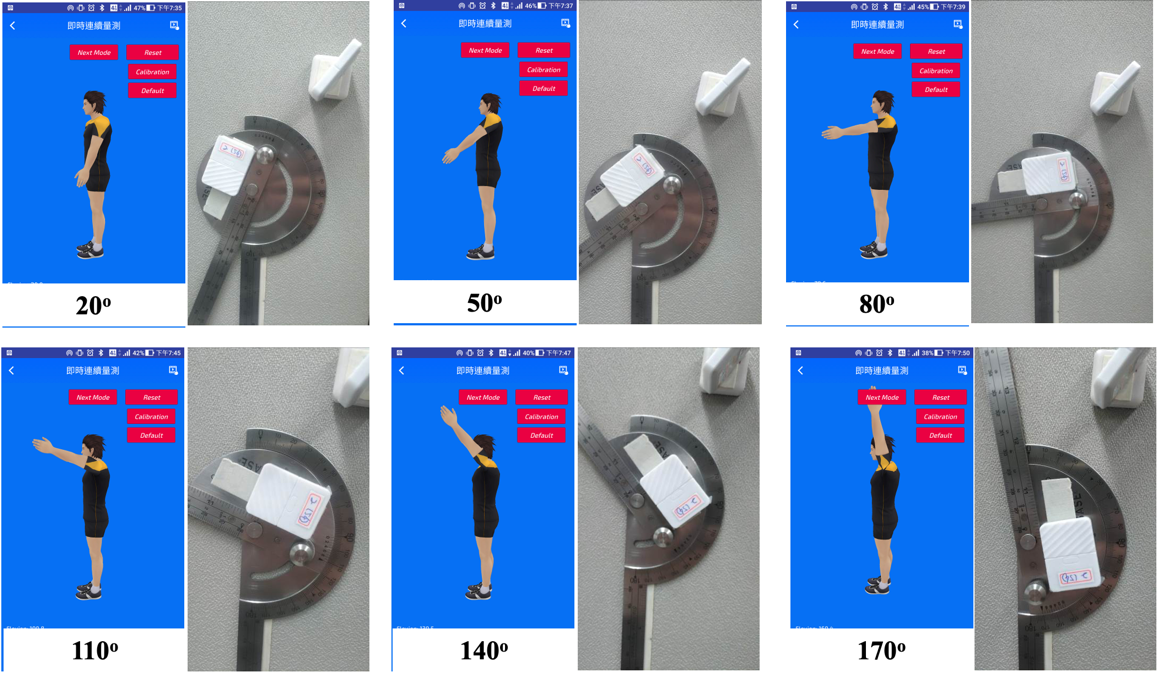


**Figure 5 Dual-sensor angular measurements on different angles**

- - 1. Upon reaching 180°, the angle of the goniometer was decreased from 180° to 0° in stages of 10° each. The angular measurements obtained by the sensor and the goniometer were recorded.
    2. Sensors S1 and S2 were replaced with sensors S3 and S4, respectively. Repeat Step 2 to Step 4.
    3. Change the tilt angle to 45° on sensors S1 and S3. Repeat Step 2 to Step 5.
  1. **Statistical analysis**

The average angular measurement errors on different axes in comparison with the angle of the goniometer were calculated and recorded.

1. **Results**
   1. **Single-sensor measurements**

| Angle on goniometer  (°) | Angle measured by sensors on **x-axis** (°) | | | | | |
| --- | --- | --- | --- | --- | --- | --- |
|  | Sensor S1 | | Sensor S1 | | Average | Measurement error |
|  | 1^st^ time | 2^nd^ time | 1^st^ time | 2^nd^ time |  |  |
| 0 | 0 | 0 | 0 | 0 | 0.00 | 0.00 |
| 30 | 30.2 | 30.1 | 30 | 30.2 | 30.13 | -0.13 |
| 60 | 60.2 | 60.4 | 60.3 | 60.3 | 60.30 | -0.30 |
| 90 | 90.4 | 90.6 | 90.4 | 90.2 | 90.40 | -0.40 |
| 120 | 120.7 | 120.6 | 120.4 | 120.5 | 120.55 | -0.55 |
| 150 | 150.4 | 150.4 | 150.5 | 150.4 | 150.43 | -0.43 |
| 180 | 180.4 | 180.4 | 180.4 | 180.4 | 180.40 | -0.40 |
| **Average error on x-axis:** | | | | | | **-0.31** |

| Angle on goniometer  (°) | Angle measured by sensors on **y-axis** (°) | | | | | |
| --- | --- | --- | --- | --- | --- | --- |
|  | Sensor S2 | | Sensor S3 | | Average | Measurement error |
|  | 1^st^ time | 2^nd^ time | 1^st^ time | 2^nd^ time |  |  |
| 0 | 0 | 0 | 0 | 0 | 0.00 | 0.00 |
| 30 | 30 | 30 | 30 | 30 | 30.00 | 0.00 |
| 60 | 60.1 | 60 | 60 | 60 | 60.03 | -0.03 |
| 90 | 90 | 89.9 | 90 | 89.8 | 89.93 | 0.08 |
| 120 | 120.2 | 120.2 | 120.2 | 120.1 | 120.18 | -0.18 |
| 150 | 150.3 | 150.2 | 150.2 | 150.2 | 150.23 | -0.23 |
| 180 | 180.3 | 180.3 | 180.4 | 180.4 | 180.35 | -0.35 |
| **Average error on y-axis:** | | | | | | **-0.10** |

| Angle on goniometer  (°) | Angle measured by sensors on **z-axis** (°) | | | | | |
| --- | --- | --- | --- | --- | --- | --- |
|  | Sensor S1 | | Sensor S1 | | Average | Measurement error |
|  | 1^st^ time | 2^nd^ time | 1^st^ time | 2^nd^ time |  |  |
| 0 | 0 | 0 | 0 | 0 | 0.00 | 0.00 |
| 30 | 30.5 | 30.3 | 30.7 | 30.6 | 30.53 | -0.53 |
| 60 | 60.8 | 60.9 | 60.9 | 60.9 | 60.88 | -0.88 |
| 90 | 90.9 | 90.9 | 91 | 91 | 90.95 | -0.95 |
| 120 | 120.8 | 120.9 | 121 | 121 | 120.93 | -0.93 |
| 150 | 150.6 | 150.7 | 150.8 | 150.9 | 150.75 | -0.75 |
| 180 | 180.4 | 180.4 | 180.3 | 180.3 | 180.35 | -0.35 |
| **Average error on z-axis:** | | | | | | **-0.63** |

- 1. **Dual-sensor measurements on a simulated shoulder movement**

| **40**° **tilt angle on fixed sensors S1 and S3** | | | | | | |
| --- | --- | --- | --- | --- | --- | --- |
| Angle on goniometer  (°) | Angular measurements (°) | | | | Average | Measurement error |
|  | Paired sensors S1 and S2 | | Paired sensors S3 and S4 | |  |  |
|  | 1^st^ time | 2^nd^ time | 1^st^ time | 2^nd^ time |  |  |
| 0 | 0 | 0 | 0 | 0 | 0.00 | 0.00 |
| 10 | 10 | 10 | 10 | 10 | 10.00 | 0.00 |
| 20 | 20 | 20 | 19.9 | 20 | 19.98 | 0.03 |
| 30 | 30 | 30.1 | 30 | 30.1 | 30.05 | -0.05 |
| 40 | 39.9 | 40.1 | 40 | 40.1 | 40.03 | -0.03 |
| 50 | 50 | 50 | 50.1 | 50 | 50.03 | -0.03 |
| 60 | 60.1 | 60.2 | 60.2 | 60 | 60.13 | -0.13 |
| 70 | 70.2 | 70.1 | 70.3 | 70 | 70.15 | -0.15 |
| 80 | 80.3 | 80.2 | 80.2 | 80.1 | 80.20 | -0.2 |
| 90 | 90.2 | 90.2 | 90.2 | 90.1 | 90.18 | -0.18 |
| 100 | 100.4 | 100.4 | 100.2 | 100.2 | 100.30 | -0.30 |
| 110 | 110.4 | 110.4 | 110.4 | 110.2 | 110.35 | -0.35 |
| 120 | 120.4 | 120.3 | 120.1 | 120.5 | 120.33 | -0.33 |
| 130 | 130.5 | 130.4 | 130.4 | 130.2 | 130.38 | -0.38 |
| 140 | 140.6 | 140.4 | 140.3 | 140.3 | 140.40 | -0.40 |
| 150 | 150.5 | 150.3 | 150.3 | 150.3 | 150.35 | -0.35 |
| 160 | 160.3 | 160.4 | 160.2 | 160.3 | 160.30 | -0.30 |
| 170 | 170.3 | 170.3 | 170.3 | 170.3 | 170.30 | -0.30 |
| 180 | 180.3 | 180.3 | 180.2 | 180.2 | 180.25 | -0.25 |
| **Average error of dual-sensor angular measurement** | | | | | | **-0.19** |

| **45**° **tilt angle on fixed sensors S1 and S3** | | | | | | |
| --- | --- | --- | --- | --- | --- | --- |
| Angle on goniometer  (°) | Angular measurements (°) | | | | Average | Measurement error |
|  | Paired sensors S1 and S2 | | Paired sensors S3 and S4 | |  |  |
|  | 1^st^ time | 2^nd^ time | 1^st^ time | 2^nd^ time |  |  |
| 0 | 0 | 0 | 0 | 0 | 0.00 | 0.00 |
| 10 | 10.2 | 10.2 | 10.1 | 10.2 | 10.18 | -0.18 |
| 20 | 20.2 | 20.2 | 20 | 19.8 | 20.05 | -0.05 |
| 30 | 30.2 | 30 | 29.9 | 29.8 | 29.98 | 0.03 |
| 40 | 40 | 40.2 | 40.1 | 40.2 | 40.13 | -0.13 |
| 50 | 50.6 | 50.4 | 50.3 | 49.9 | 50.30 | -0.30 |
| 60 | 60.6 | 60.4 | 60.3 | 60.5 | 60.45 | -0.45 |
| 70 | 70.5 | 70.4 | 70.4 | 70.2 | 70.38 | -0.38 |
| 80 | 80.5 | 80.4 | 80.5 | 80.4 | 80.45 | -0.45 |
| 90 | 90.3 | 90.3 | 90.5 | 90.6 | 90.43 | -0.43 |
| 100 | 100.6 | 100.4 | 100.3 | 100.5 | 100.45 | -0.45 |
| 110 | 110.6 | 110.5 | 110.5 | 110.5 | 110.53 | -0.53 |
| 120 | 120.4 | 120.5 | 120.4 | 120.5 | 120.45 | -0.45 |
| 130 | 130.3 | 130.4 | 130.2 | 130.2 | 130.28 | -0.28 |
| 140 | 140.2 | 140.1 | 140 | 140.3 | 140.15 | -0.15 |
| 150 | 150.1 | 150.3 | 150.2 | 149.9 | 150.13 | -0.13 |
| 160 | 160.1 | 160.1 | 159.6 | 159.7 | 159.88 | 0.13 |
| 170 | 170 | 170.1 | 169.8 | 170 | 169.98 | 0.03 |
| 180 | 180 | 180 | 179.6 | 179.6 | 179.80 | 0.20 |
| **Average error of dual-sensor angular measurement** | | | | | | **-0.21** |

1. **Conclusions**

The present findings revealed that both single-sensor and dual-sensor systems for angular measurement had high accuracy and consistency when compared with the angles reported on the goniometer. Both systems meet the pre-set specification of angular measurement errors of ± 1°.
