# Supplementary material for: Wearable Motion Sensor Device to Facilitate Rehabilitation in Patients With Shoulder Adhesive Capsulitis: Pilot Study to Assess Feasibility
Source: J Med Internet Res. 2020 Jul 23;22(7):e17032. doi: 10.2196/17032 (PMC7413285; doi:10.2196/17032)
Supplement: Multimedia Appendix 3 [file jmir_v22i7e17032_app3.docx]

**Home-based Exercise Protocol for Shoulder Adhesive Capsulitis**

This is the standard home-based exercise protocol for shoulder adhesive capsulitis for all participants in our study. The protocol includes two parts: Warm-Up and Stretches. For each stretching exercise, 10-10 rule (10 times each exercise per day, each exercise lasting at least 10 seconds) is suggested for all participants. Patients are encouraged to apply a heating pad on the involved shoulder for at least 10 minutes before exercise. The whole exercise program will take around 40 minutes.

**Warm-up**

**Shoulder Pendulum Exercise**

***
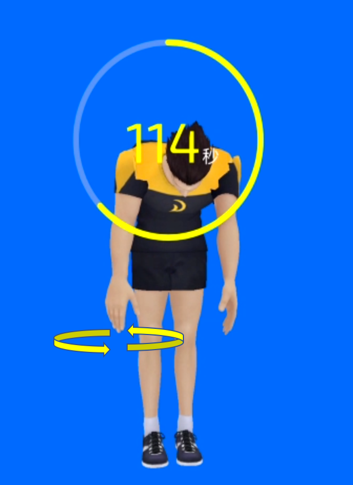
Description:***

- Lean over with your uninvolved arm supported on a table or chair.
- Relax and hang the involved arm straight down
- Move slowly the involved arm in a circle, then reverse the direction. Next, move the arm backward and forward. Finally, move it side to side.

***Frequency:***

Warm up for 5 to 10 minutes before the stretching exercise. Change the direction of your movement after 1 minute of motion.

**Shoulder Stretch**

**Forward wall walking stretch**


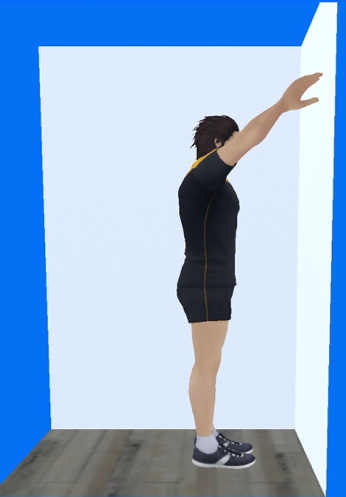
***Description:***

- Patient stands facing the wall
- Walk fingers on the involved side slowly up the wall to as high as possible, pulling the arm upwards.
- Hold at the top for at least 10 seconds, then lower the arm slowly

***Frequency:***

10 times per day

**Lateralward wall walking stretch**

**
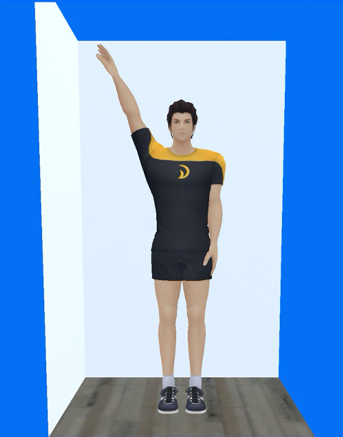
*Description:***

- Patient stands with their involved shoulder 1 meter from the wall
- Walk fingers on the involved side slowly up the wall to as high as possible, pulling the arm upwards.
- Hold at the top for at least 10 seconds, then lower the arm slowly

***Frequency:***

10 times per day

**Cane stretch for shoulder flexion**


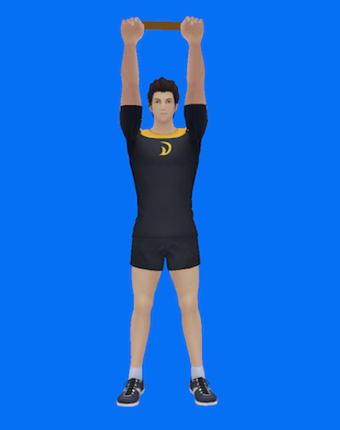
***Description:***

- Grasp a wand horizontally on both ends
- Led by uninvolved side, raise the wand slowly above the head until a stretch is felt
- Hold the position for at least 10 seconds, then return to starting position

***Frequency:***

10 times per day

**Cane stretch for shoulder abduction**


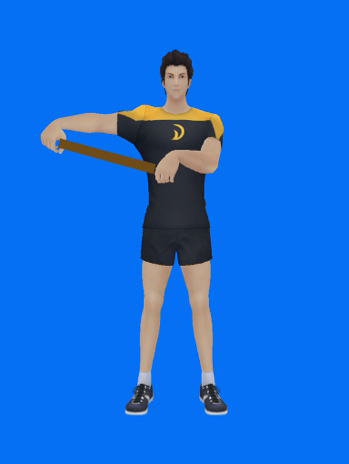
***Description:***

- Grasp a wand upright with the hand of the involved side up and the hand of the uninvolved side down
- With the hand of the uninvolved side, push the wand directly out from your side until a stretch is felt
- Hold the position for at least 10 seconds and return to starting position

***Frequency:***

10 times per day

**Cane stretch for shoulder external rotation**

**
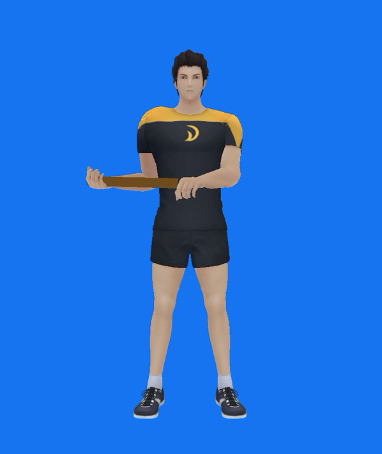
*Description:***

- Hold a wand (with the back leaning against a wall), palm of the involved side facing up and palm of the uninvolved side facing down
- Push the wand to the side of the involved shoulder and let the involved shoulder roll outward until a stretch is felt
- Hold the stretch for at least 10 seconds and return to starting position

***Frequency:***

10 times per day

**Cane stretch for shoulder internal rotation**

***
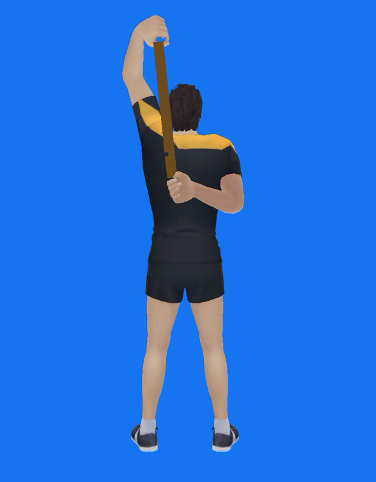
Description:***

- Hold a wand behind the back with the hand of the uninvolved side up and the hand of the involved side down
- With the hand of the uninvolved side, pull the wand upward until a stretch is felt
- Hold the stretch for at least 10 seconds and return to starting position

***Frequency:***

10 times per day

**Cane stretch for shoulder extension**

***
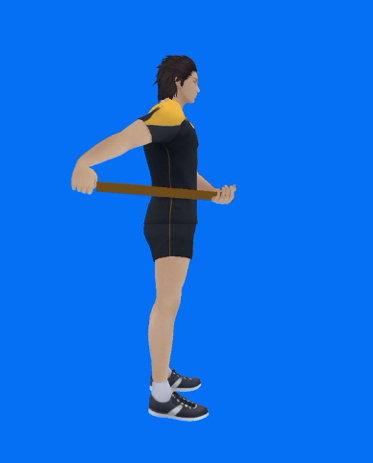
Description:***

- Grasp a wand upright with the hand of the involved side down and the hand of the uninvolved side up
- With the hand of the uninvolved side, push the hand of the involved side backward until a stretch is felt
- Hold the position for at least 10 seconds and return to starting position

***Frequency:***

10 times per day
