# Supplementary material for: Wearable Motion Sensor Device to Facilitate Rehabilitation in Patients With Shoulder Adhesive Capsulitis: Pilot Study to Assess Feasibility
Source: J Med Internet Res. 2020 Jul 23;22(7):e17032. doi: 10.2196/17032 (PMC7413285; doi:10.2196/17032)
Supplement: Multimedia Appendix 4 [file jmir_v22i7e17032_app4.docx]

**Appendix Table 1:** Correlation between changes in qDASH score and shoulder ROM in the different directions at different time points of follow-up from baseline

| **Spearman correlation between angular change of shoulder ROM and change in qDash score from baseline** **(R)** | | | | | | | | | | |
| --- | --- | --- | --- | --- | --- | --- | --- | --- | --- | --- |
| Follow-up time points | Abduction | | Flexion | | Extension | | External rotation | | Internal rotation | |
|  | Active | Passive | Active | Passive | Active | Passive | Active | Passive | Active | Passive |
| 1 month | -0.525 | -0.394 | -0.194 | -0.374 | -0.341 | -0.271 | -0.365 | -0.313 | -0.451 | -0.539* |
| 2 months | -0.216 | -0.415 | -0.191 | -0.374 | -0.457 | -0.374 | -0.348 | -0.260 | -0.254 | -0.404 |
| 3 months | -0.484 | -0.493 | -0.273 | -0.407 | -0.491 | -0.522 | -0.283 | -0.211 | -0.250 | -0.202 |

**Abbreviation:** ROM: range of motion, qDASH: Quick Disabilities of the Arm, Shoulder, and Hand questionnaire

*: p < 0.05
